# Nanoencapsulated *Dunaliella tertiolecta* Extract and β-Carotene in Liposomal Carriers: Antioxidant and Erythroprotective Potential Through Sustained-Release Systems

**DOI:** 10.3390/molecules30193924

**Published:** 2025-09-29

**Authors:** Jonathan García-Morales, Ricardo Iván González-Vega, Diana Fimbres-Olivarría, Ariadna Thalía Bernal-Mercado, Santiago Pedro Auobourg-Martínez, Karla Alejandra López-Gastélum, Silvia Elena Burruel-Ibarra, María Irene Silvas-García, Andrea Grijalva-Molina, José de Jesús Ornelas-Paz, Carmen Lizette Del-Toro-Sánchez

**Affiliations:** 1 Department of Research and Postgraduate in Food, University of Sonora, Blvd Luis Encinas y Rosales S/N, Col. Centro, Hermosillo 83000, SO, Mexicokarla.lopezgastelum@unison.mx (K.A.L.-G.);; 2 Department of Health Sciences, University Center of the Valleys (CUVALLE), University of Guadalajara, Carr. a Guadalajara Km. 45.5, Ameca 46600, JA, Mexico; 3 Departamento de Investigaciones Científicas y Tecnológicas, Universidad de Sonora, Blvd Luis Donaldo Colosio W/N, Hermosillo 83000, SO, Mexico; diana.fimbres@unison.mx; 4 Departamento de Tecnología en Alimentos, Instituto de Investigaciones Marinas (CSIC). Av. Eduardo Cabello No. 6, Vigo, 36208 Penevedra, Spain; 5 Departamento de Investigación en Polímero y Materiales, Universidad de Sonora, Blvd. Luis Encinas y Rosales S/N, Col. Centro, Hermosillo 83000, SO, Mexico; 6 Centro de Investigación en Alimentación y Desarrollo A.C.-Unidad Cuauhtémoc, Av. Río Conchos S/N, Parque Industrial, Ciudad Cuauhtémoc 31570, CUU, Mexico

**Keywords:** *Dunaliella tertiolecta*, β-carotene, nanoliposomes, nanoencapsulation, antioxidant activity, erythroprotective effect, sustained release, oxidative stress

## Abstract

The nanoencapsulation of bioactive compounds such as β-carotene and microalgal extracts has emerged as an effective strategy to enhance their stability, bioavailability, and biological efficacy, particularly against oxidative stress. *Dunaliella tertiolecta*, a microalga rich in carotenoids and chlorophylls, presents notable antioxidant and erythroprotective properties; however, its bioactive potential is limited by low bioaccessibility and degradation during processing and digestion. This study aimed to develop and evaluate nanoliposomes loaded with *D. tertiolecta* extract and β-carotene as sustained-release systems to improve antioxidant performance and erythroprotective effects. The methodology involved optimizing microalgal cultivation under nitrogen and salinity stress to enhance pigment accumulation, followed by extraction, nanoencapsulation via the particle dispersion method, and physicochemical characterization of the nanoliposomes. Antioxidant capacity and release kinetics were assessed through ABTS and FRAP assays, while erythroprotective activity was evaluated by monitoring oxidative hemolysis in human erythrocytes. The release kinetics revealed an anomalous transport mechanism for both systems, with β-carotene showing faster and more efficient release due to its greater lipophilic compatibility with the nanoliposomal matrix. The nanoliposomal systems demonstrated nanoscale size, high encapsulation efficiency, sustained antioxidant release, and effective erythrocyte protection, with the extract-loaded formulation exhibiting synergistic effects superior to isolated β-carotene. These findings support the potential application of this nanotechnology-based delivery system in functional foods, nutraceuticals, and biomedical formulations aimed at preventing oxidative stress-related cellular damage.

## 1. Introduction

Oxidative stress, characterized by an imbalance between the production of reactive oxygen species (ROS) and the antioxidant defense mechanisms of the organism, plays a central role in the pathogenesis of various chronic and degenerative diseases, including cardiovascular disorders, diabetes, anemia, and cancer [1,2]. At the molecular level, ROS such as superoxide anion (O_2_•^−^), hydroxyl radical (•OH), and hydrogen peroxide (H_2_O_2_) induce lipid peroxidation, protein oxidation, and DNA damage, compromising cellular integrity and function [3,4,5,6]. Antioxidant molecules counteract these deleterious effects through mechanisms including hydrogen atom transfer (HAT), where hydrogen atoms are donated to neutralize free radicals, and single electron transfer (SET), where electrons are donated to stabilize reactive species [7,8,9]. Carotenoids such as β-carotene and chlorophyll derivatives exert potent antioxidant effects by quenching singlet oxygen (^1^O_2_) and scavenging peroxyl radicals, contributing to membrane stabilization and protection of cellular structures [10,11]. However, the efficacy of these compounds is limited by their hydrophobic nature, low aqueous solubility, and susceptibility to oxidative degradation during processing and gastrointestinal digestion.

In recent years, nanoencapsulation technologies have emerged as powerful tools to improve the stability, bioavailability, and functionality of bioactive compounds. Among these, nanoliposomes—nanoscale vesicles composed of phospholipid bilayers—offer significant advantages as delivery systems due to their amphiphilic architecture, which enables the encapsulation of both hydrophobic and hydrophilic molecules [12,13]. This structural arrangement protects sensitive compounds from environmental factors such as light, oxygen, and pH fluctuations, while promoting controlled and sustained release [14,15,16]. Prior studies have successfully employed nanoliposomal encapsulation for various carotenoids, including fucoxanthin and astaxanthin, demonstrating improvements in their antioxidant activity, bioaccessibility, and cellular uptake. Furthermore, microalgal extracts from species such as *Haematococcus pluvialis*, *Chlorella vulgaris*, and *Spirulina platensis* have been incorporated into nanocarriers, enhancing their functional performance [17,18,19]. However, despite these advances, the encapsulation of *Dunaliella tertiolecta* extracts, rich in a diverse pool of carotenoids and chlorophylls, remains poorly explored, particularly in systems designed for sustained release and erythroprotective applications.

To date, no studies have comprehensively addressed the nanoencapsulation of *D. tertiolecta* extract and β-carotene in nanoliposomal carriers for the dual purpose of antioxidant delivery and erythrocyte membrane protection against oxidative damage. The nanoencapsulation of carotenoids into nanoliposomes enhances their chemical stability, protects them from oxidative degradation, and improves their solubility and bioavailability in biological systems. This encapsulation strategy enables the sustained and controlled release of the bioactive compounds, allowing a gradual interaction with reactive oxygen species and prolonging their antioxidant action over time. By maintaining effective concentrations of carotenoids at the target site, nanoliposomes optimize the scavenging of free radicals and support continuous membrane protection [20,21,22]. This prolonged release profile is crucial to maximize the therapeutic potential of carotenoids in preventing oxidative stress-related cellular damage [23]. This represents a significant gap in the current literature, as previous research has focused primarily on isolated carotenoids or other microalgal species without integrating synergistic pigment mixtures in encapsulation strategies [18,19].

In this study, the innovative contribution does not rely solely on the development of nanoliposomes encapsulating *Dunaliella tertiolecta* extract and β-carotene, but also on the optimization of the extraction process itself, which represents a critical step to maximize the bioactivity of microalgal compounds before encapsulation. Cultivation of *D. tertiolecta* under nitrogen and salinity stress was strategically optimized to enhance the accumulation of carotenoids and chlorophylls, thereby improving the quality of the bioactive extract. This approach highlights the novelty of integrating upstream microalgal cultivation optimization with downstream nanoencapsulation, ensuring that the bioactive potential of complex mixtures of carotenoids and xanthophylls is preserved and effectively delivered. By linking the optimization of pigment accumulation with the design of nanoliposomal carriers, this research provides a dual innovation: (i) an enhanced extract rich in structurally diverse compounds with antioxidant and erythroprotective properties, and (ii) its stabilization and functional delivery through sustained-release liposomal systems. This integrative strategy addresses a major bottleneck in microalgal biotechnology, where bioaccessibility and degradation often compromise efficacy, and opens new opportunities for the design of functional foods and nutraceuticals targeting oxidative stress-related disorders.

The main objective of this study was to develop, characterize, and compare nanoliposomes individually loaded with *Dunaliella tertiolecta* extract or pure β-carotene, focusing on their physicochemical properties, antioxidant capacity, release kinetics, and erythroprotective potential under oxidative stress conditions. By analyzing and contrasting the performance of these two formulations, this work provides insights into how the compositional complexity of natural extracts versus isolated carotenoids influences encapsulation behavior, stability, and bioactivity within sustained-release liposomal systems. In a broader perspective, these findings contribute to the advancement of nanotechnology-based delivery platforms with potential applications in the design of functional foods, nutraceuticals, and biomedical formulations aimed at mitigating oxidative stress-related cellular damage and supporting long-term health.

## 2. Results and Discussions

### 2.1. Growth Kinetics of Dunaliella tertiolecta

The growth kinetics of microalgae provide valuable insights into their cellular proliferation dynamics and metabolic responses under varying environmental conditions. In this study, the growth behavior of *Dunaliella tertiolecta* was systematically evaluated over 21 days under different nitrogen concentrations (0.88, 0.44, and 0.22 mol·L^−1^ of NaNO_3_) and salinity levels (25, 35, and 45 PSU). Cell density, expressed as Log_2_ cell/mL, was monitored daily to characterize the distinct growth phases, including lag, exponential, and stationary stages. This approach allowed for the assessment of how nutrient availability and osmotic stress influence the growth performance of *D. tertiolecta*, providing a basis for understanding the optimal conditions required to maximize biomass accumulation and potential pigment production. The following sections describe and discuss in detail the growth kinetics observed under each experimental condition.

**Figure 1:**
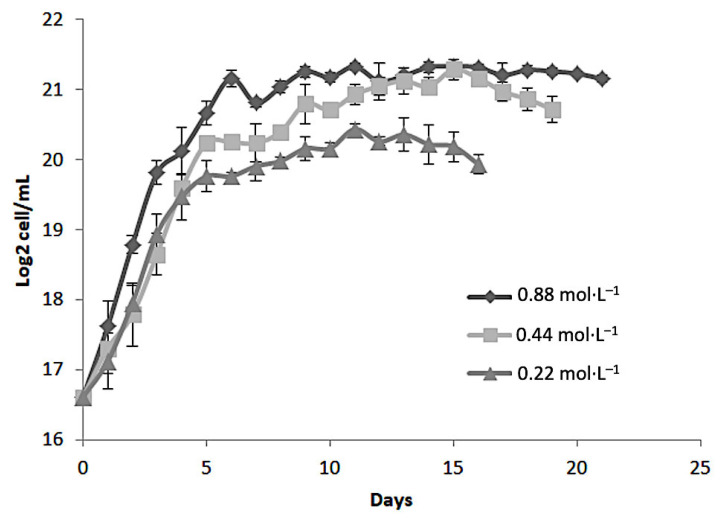
Growth kinetics (log2 cell/mL) of the microalgae *Dunaliella tertiolecta* at 25 PSU in different nitrogen concentrations (0.88 mol·L^−1^, 0.44 mol·L^−1^and 0.22 mol·L^−1^ of NaNO_3_).

Figure 1 shows the growth kinetics of *Dunaliella tertiolecta*, expressed as Log_2_ cell/mL, cultivated at 25 PSU under three different nitrogen concentrations: 0.88 mol·L^−1^, 0.44 mol·L^−1^, and 0.22 mol·L^−1^ of NaNO_3_. The cultures were monitored over 21 days to evaluate the influence of nitrogen availability on cell proliferation. The results demonstrate a clear dependency of microalgal growth on nitrogen concentration. The highest cell density was consistently observed in cultures supplemented with 0.88 mol·L^−1^ NaNO_3_, indicating that nitrogen sufficiency strongly promotes cellular proliferation and biomass accumulation in *D. tertiolecta*. Cultures at this concentration exhibited a rapid exponential growth phase within the first 5 to 7 days, reaching a peak of approximately 21 Log_2_ cell/mL, followed by a stationary phase that remained stable until the end of the experiment. In contrast, the intermediate nitrogen concentration (0.44 mol·L^−1^) also supported robust growth, though the maximum cell density achieved was slightly lower than that observed at 0.88 mol·L^−1^. The growth curve displayed a similar exponential trend but plateaued at around 20 Log_2_ cell/mL. This suggests that while 0.44 mol·L^−1^ NaNO_3_ is sufficient to sustain cell division and biomass production, it may not fully meet the nitrogen demands required for maximal proliferation under the given culture conditions.

The lowest nitrogen concentration tested (0.22 mol·L^−1^ NaNO_3_) led to the poorest growth performance. Cultures at this level showed a shortened exponential phase, reaching a maximum of ~19 Log_2_ cell mL^−1^, with an earlier onset of the stationary phase compared to other treatments. This early arrest is attributed to nitrogen limitation, which restricts protein synthesis, nucleic acid metabolism, and chlorophyll production, ultimately inhibiting cell division and biomass accumulation. These results align with previous studies emphasizing nitrogen’s essential role in microalgal growth and metabolism [24,25]. As a key macronutrient, nitrogen is involved in the synthesis of amino acids, nucleotides, and pigments like chlorophylls and carotenoids, which are vital for photosynthesis and cellular function [26]. The observed growth differences highlight the need to optimize nitrogen levels to boost microalgal productivity, especially when aiming to maximize biomass or secondary metabolite production, such as antioxidant pigments [24,25,26,27].

Overall, the growth behavior of *D. tertiolecta* under varying nitrogen regimes suggests that 0.88 mol·L^−1^ NaNO_3_ provides the most favorable conditions for cell proliferation and biomass accumulation. However, suboptimal nitrogen levels not only limit growth but may also trigger metabolic shifts toward the accumulation of stress-related secondary metabolites, such as carotenoids, which could be beneficial depending on the intended application [28].

**Figure 2:**
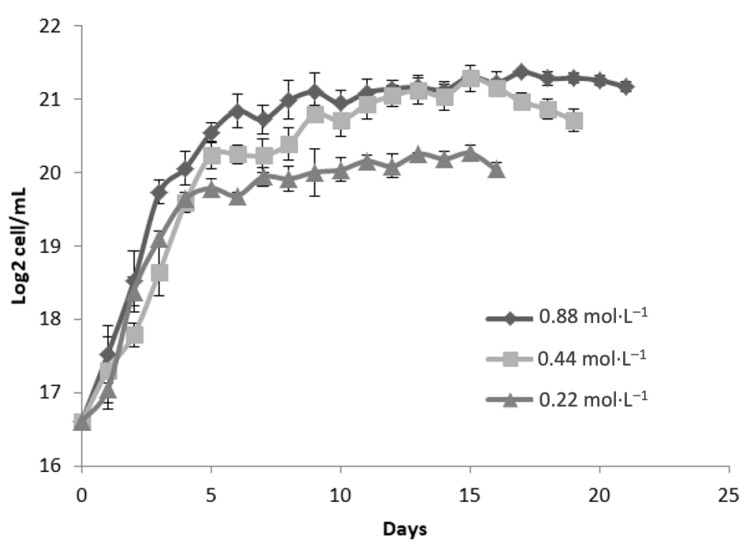
Growth kinetics (log2 cell/mL) of the microalgae *Dunaliella tertiolecta* at 35 PSU in different nitrogen concentrations (0.88 mol·L^−^^1^, 0.44 mol·L^−^^1^ and 0.22 mol·L^−^^1^ of NaNO_3_).

Figure 2 illustrates the growth kinetics of *Dunaliella tertiolecta*, represented as Log_2_ cell/mL, cultivated at 35 PSU under three nitrogen regimes: 0.88 mol·L^−1^, 0.44 mol·L^−1^, and 0.22 mol·L^−1^ of NaNO_3_. The cultures were monitored for 21 days to assess the impact of nitrogen concentration on the microalgal proliferation at elevated salinity conditions. The results indicate that nitrogen availability significantly influences the growth performance of *D. tertiolecta* at 35 PSU. The culture supplemented with the highest nitrogen concentration (0.88 mol·L^−1^ NaNO_3_) achieved the greatest cell density, reaching approximately 21 Log_2_ cell mL^−^^1^ during the stationary phase [29]. Similarly to the behavior observed at 25 PSU, this treatment exhibited a rapid exponential growth phase within the initial 5 to 7 days, followed by stabilization of cell density, suggesting that sufficient nitrogen supply supports sustained biomass accumulation even under higher salinity stress [30].

Cultures grown with 0.44 mol·L^−1^ NaNO_3_ showed similar growth kinetics during the exponential phase, though the maximum cell concentration was slightly lower than that of the 0.88 mol·L^−1^ treatment. This suggests that moderate nitrogen levels support cell proliferation but may not meet the full nitrogen demands for optimal biomass under saline conditions. In contrast, cultures with 0.22 mol·L^−1^ NaNO_3_ exhibited significantly lower growth rates and reached the lowest cell density (~19 Log_2_ cell/mL), with an earlier stationary phase. This reflects nitrogen limitation, which restricts key biosynthetic processes such as protein, nucleic acid, and pigment synthesis [25].

Comparing growth at 35 PSU with 25 PSU (Figure 1), a slight decline in maximum cell densities was observed across all nitrogen levels, suggesting that increased salinity imposes additional osmotic stress, affecting cell homeostasis and metabolism. Nevertheless, nitrogen supplementation at 0.88 mol·L^−1^ appears to mitigate these effects, supporting sustained growth. These patterns highlight the essential role of nitrogen in maintaining cell function and biomass under saline stress. As a key nutrient in the biosynthesis of essential components, nitrogen limitation disrupts both cell proliferation and metabolic pathways tied to stress responses and secondary metabolite production. Elevated salinity further increases nitrogen demand due to the energy required for osmoregulation and compatible solute synthesis [25,29,30,31].

**Figure 3:**
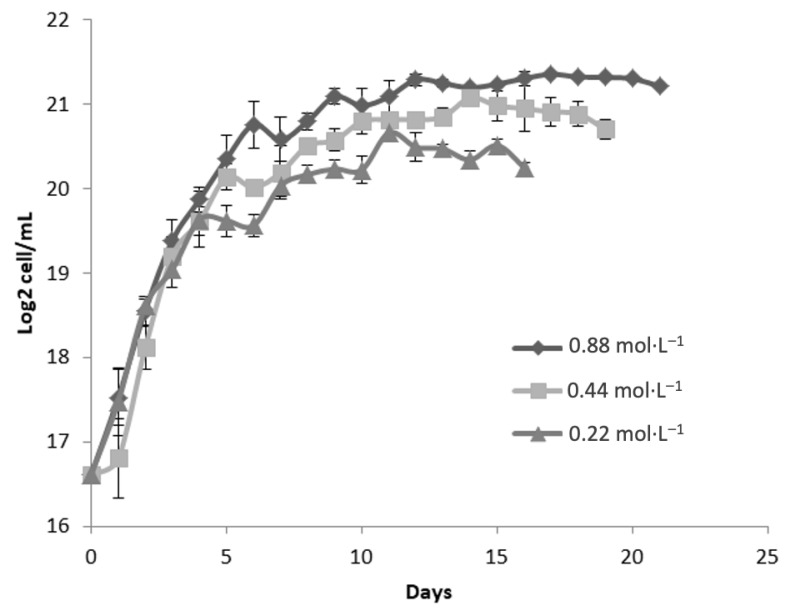
Growth kinetics (log2 cell/mL) of the microalgae *Dunaliella tertiolecta* at 45 PSU in different nitrogen concentrations (0.88 mol·L^−^^1^, 0.44 mol·L^−^^1^ and 0.22 mol·L^−^^1^ of NaNO_3_).

Figure 3 presents the growth kinetics of *Dunaliella tertiolecta*, expressed as Log_2_ cell/mL, cultivated at a salinity of 45 PSU with three nitrogen concentrations: 0.88 mol·L^−1^, 0.44 mol·L^−1^, and 0.22 mol·L^−1^ of NaNO_3_. The culture performance was evaluated over 21 days to determine the effect of nitrogen availability on microalgal proliferation under high-salinity stress conditions. The results demonstrate that nitrogen concentration exerts a significant influence on cell growth, even under elevated salinity. The highest nitrogen concentration (0.88 mol·L^−1^ NaNO_3_) consistently supported the greatest biomass accumulation, achieving a maximum of approximately 21 Log_2_ cell/mL. This treatment exhibited a well-defined exponential growth phase during the first 5 to 7 days, followed by the onset of the stationary phase, where cell densities remained stable until the end of the experimental period.

At the intermediate nitrogen level (0.44 mol·L^−1^ NaNO_3_), a similar growth trend was observed, though the maximum cell concentration was slightly lower than that of the 0.88 mol·L^−1^ treatment. This suggests that moderate nitrogen supports cell proliferation but may not fully meet the elevated metabolic demands under high salinity. The lowest nitrogen concentration (0.22 mol·L^−1^ NaNO_3_) resulted in the poorest performance, with cultures reaching only ~19 Log_2_ cell/mL and an earlier transition to the stationary phase—signs of severe nitrogen limitation. The restricted nitrogen supply, combined with osmotic stress at 45 PSU, likely increased the metabolic burden, impairing protein synthesis, chlorophyll production, and division. Compared to 25 and 35 PSU (Figure 1 and Figure 2), a progressive reduction in cell density was observed across all nitrogen levels as salinity increased. This trend underscores the negative impact of osmotic pressure on growth, as cells divert metabolic resources toward stress adaptation mechanisms like glycerol synthesis [25].

Despite the negative impact of high salinity on overall biomass yield, nitrogen supplementation at 0.88 mol·L^−^^1^ appears to mitigate, at least partially, the growth inhibition associated with saline stress. This suggests that adequate nitrogen availability is essential not only for supporting basic metabolic functions but also for enabling osmoregulatory responses under extreme environmental conditions. These findings reinforce the critical role of nitrogen nutrition in the physiological performance of *D. tertiolecta*, particularly under high-salinity cultivation strategies commonly employed for the production of carotenoids and other high-value metabolites. Nitrogen limitation, while detrimental to biomass accumulation, is known to trigger the accumulation of secondary metabolites such as β-carotene due to stress-induced metabolic shifts. Therefore, the deliberate modulation of nitrogen supply in combination with salinity control could be strategically used to optimize either biomass production or metabolite enrichment, depending on the desired biotechnological outcome [32].

Growth kinetics analysis of *Dunaliella tertiolecta* under varying nitrogen and salinity conditions confirmed that both significantly affect cell proliferation and biomass [33]. The best outcome was observed at 25 PSU with 0.88 mol·L^−1^ NaNO_3_, where cultures entered stationary phase by day 7, maintaining the highest cell density. This suggests that harvesting at day 7 under these conditions is optimal for maximizing yield before nutrient depletion or loss of viability. These findings offer a valuable reference for optimizing *D. tertiolecta* cultivation when high biomass productivity is the target [26,33].

### 2.2. Optimizing Key Factors for the Biosynthesis of Chlorophyll a, b, and c

The optimization of chlorophyll pigment production in *Dunaliella tertiolecta* was successfully evaluated through a Central Composite Design (CCD) matrix, considering nitrogen concentration (X_1_), salinity (X_2_), and culture age (X_3_) as the independent variables. The responses analyzed included the concentrations of chlorophyll a (Cₐ), chlorophyll b (C_b_), and chlorophyll c (C_c1+c2_), expressed in mg/g of dry weight (dw). Both experimental responses and the predicted values obtained from the statistical model are presented in Table 1.

The results demonstrated significant variability in pigment production across the different experimental runs, highlighting the crucial influence of the tested factors and their interactions. The most remarkable pigment accumulation was observed in Run 10, where the nitrogen concentration was at its lowest level (0.22 mol·L^−1^), salinity at 25 PSU, and culture age at 15 days. Under these conditions, the maximum experimental values for chlorophyll a (13.68 mg/g dw), chlorophyll b (4.85 mg/g dw), and chlorophyll c (3.71 mg/g dw) were recorded. The predicted responses by the model in this condition also showed high agreement with the experimental data (12.29 mg/g dw for Cₐ, 4.92 mg/g dw for C_b_, and 3.41 mg/g dw for C_c1+c2_), confirming the model’s reliability and robustness in describing pigment production behavior under these specific conditions.

The combination of low nitrogen availability and lower salinity appears to favor pigment accumulation, especially in later stages of cultivation. This is likely due to stress-induced metabolic responses in microalgae, where nitrogen limitation triggers carotenoid and chlorophyll synthesis as part of photoprotective and adaptive mechanisms [28,34]. In contrast, lower pigment levels were observed under higher nitrogen and salinity conditions at early harvest times. For instance, Run 2 (0.55 mol·L^−1^ nitrogen, 35 PSU, 5 days) showed significantly reduced chlorophyll yields (1.0 mg/g dw for Cₐ, 0.73 mg/g dw for C_b_, 0.12 mg/g dw for C_c1+c2_). These results suggest that nitrogen sufficiency and short culture durations favor biomass growth over secondary metabolite production under non-stress conditions [35].

The model’s predictive performance was validated by a non-significant lack-of-fit test (*p* > 0.05), confirming the adequacy of the quadratic polynomial equation in describing the relationship between factors and response variables. The close match between experimental and predicted values across most runs further supports the suitability of the CCD approach and the effectiveness of Response Surface Methodology (RSM) in modeling complex interactions. Data trends confirmed that nitrogen concentration, salinity, and culture age significantly influence pigment biosynthesis in *D. tertiolecta*. Specifically, nitrogen limitation combined with low to moderate salinity and extended culture duration proved most favorable for maximizing pigment yield. These findings offer valuable guidance for designing cultivation protocols to enhance pigment production in microalgae-based systems. Overall, the CCD, supported by RSM analysis, proved effective for identifying optimal conditions and understanding environmental interactions affecting chlorophyll biosynthesis. This approach provides a solid framework for future applications in microalgal biotechnology, particularly for high-value pigment production [36,37].

The statistical evaluation of chlorophyll pigment production in *Dunaliella tertiolecta* using a Central Composite Design revealed distinct effects of nitrogen concentration, salinity, and light intensity, as well as their interactions, on the biosynthesis of chlorophylls a, b, and c. Analysis of variance (ANOVA) focused on the F value and significance level (Prob > F) to assess each factor’s contribution to pigment accumulation. For chlorophyll a, nitrogen concentration (X_2_) was the most significant factor (F = 10.9196, Prob > F = 0.0163 **), indicating a clear effect at *p* < 0.05. The nitrogen–light interaction (X_1_ × X_3_) also showed significance (F = 9.1295, Prob > F = 0.0234 **), while the nitrogen–salinity interaction (X_1_ × X_2_) had a highly significant effect (F = 23.8088, Prob > F = 0.0028 *). These results underscore the importance of nitrogen availability and its interactions with salinity and light in modulating chlorophyll a biosynthesis in *D. tertiolecta*.

For chlorophyll b production, nitrogen concentration (X_2_) was again the most influential factor, with a F value of 18.6571 and a highly significant Prob > F of 0.0050 *. Significant quadratic effects were observed for both nitrogen (X_2_ × X_2_; F = 9.8371, Prob > F = 0.0202 **) and salinity (X_1_ × X_1_; F = 11.8230, Prob > F = 0.0138 **), indicating a nonlinear response. The nitrogen–salinity interaction (X_1_ × X_2_) was highly significant (F = 61.6937, Prob > F = 0.0002 *), confirming their synergistic role in regulating chlorophyll b levels. Additionally, the nitrogen–light interaction (X_2_ × X_3_) showed substantial significance (F = 24.6091, Prob > F = 0.0026 *), suggesting that light modulates nitrogen’s effect on chlorophyll b synthesis, likely via its role in photosystem regulation and nitrogen assimilation. For chlorophyll c, nitrogen concentration (X_2_) remained a key determinant, with a F value of 12.4534 and a significant Prob > F of 0.0124 **. The nitrogen–salinity interaction (X_1_ × X_2_) had the strongest effect (F = 27.0237, Prob > F = 0.0020 *), highlighting its consistent importance across all chlorophyll types. Significant quadratic effects were also observed for salinity (X_1_ × X_1_; F = 6.7666, Prob > F = 0.0406 **) and the nitrogen–light interaction (X_2_ × X_3_; F = 7.2497, Prob > F = 0.0359 **), indicating a nonlinear response of chlorophyll c production to these environmental factors.

Overall, the ANOVA results across all three pigments consistently identify nitrogen concentration as the main factor driving chlorophyll biosynthesis in *D. tertiolecta*. However, significant interactions, particularly between nitrogen and salinity and nitrogen and light, emphasize the relevance of combined environmental modulation over isolated effects. The significant quadratic terms indicate a complex, nonlinear relationship likely involving metabolic feedback and stress adaptation mechanisms. These findings align with the previously discussed growth kinetics, where higher nitrogen levels under optimal salinity favored biomass accumulation, providing a physiological basis for increased chlorophyll synthesis. This integrated analysis supports the targeted adjustment of cultivation conditions to enhance pigment production, with promising applications in nutraceutical and biotechnological fields [38].

To support these findings and enable predictive modeling of pigment synthesis under varying culture conditions, mathematical modeling and statistical optimization were applied. Second-order polynomial equations were developed to more accurately estimate chlorophyll production and visualize factor interactions using response surface methodology. Predictive models for chlorophyll a, b, and c (Equations (1)–(3)) were generated with JMP V18 software, allowing estimation of pigment yields and construction of response surface plots. The predicted production ranges were 0.66–13.68 mg/g dw for chlorophyll a, 0.48–4.85 mg/g dw for chlorophyll b, and 0.12–3.71 mg/g dw for chlorophyll c.

6.6687183908046 + (−1.8939393939394 × x_1_) + (−0.1559 × x_2_) + (0.0226 × x_3_) + ((x_1_ − 0.55) × ((x_1_ − 0.55) × 13.4083784554004)) + ((x_1_ − 0.55) × ((x_2_ − 35) × 0.77992424242424)) + ((x_2_ − 35) × ((x_2_ − 35) × 0.01870172413793)) + ((x_1_ − 0.55) × ((x_3_ − 10) × −0.9659090909091)) + ((x_2_ − 35) × ((x_3_ − 10) × −0.024175)) + ((x_3_ − 10) × ((x_3_ − 10) × 0.03560689655172)) (1)

2.54573563218391 + (0.22424242424242 × x_1_) + (−0.0514 × x_2_) + (−0.0418 × x_3_) + ((x_1_ − 0.55) × ((x_1_ − 0.55) × 7.317691016675058)) + ((x_1_ − 0.55) × ((x_2_ − 35) × 0.31666666666667)) + ((x_2_ − 35) × ((x_2_ − 35) × 0.00726896551724)) + ((x_1_ − 0.55) × ((x_3_ − 10) × −0.4348484848485)) + ((x_2_ − 35) × ((x_3_ − 10) × −0.0132)) + ((x_3_ − 10) × ((x_3_ − 10) × 0.01167586206897)) (2)

1.90072413793104 + (−0.4272727272727 × x_1_) + (−0.0444 × x_2_) + (0.004 × x_3_) + ((x_1_ − 0.55) × ((x_1_ − 0.55) × 5.85320287514645)) + ((x_1_ − 0.55) × ((x_2_ − 35) × 0.22159090909091)) + ((x_2_ − 35) × ((x_2_ − 35) × 0.00382413793103)) + ((x_1_ − 0.55) × ((x_3_ − 10) × −0.2522727272727)) + ((x_2_ − 35) × ((x_3_ − 10) × −0.007575)) + ((x_3_ − 10) × ((x_3_ − 10) × 0.00409655172414)) (3)

The second-order polynomial equations for chlorophyll a, b, and c production reveal how nitrogen concentration (x_1_), salinity (x_2_), and light intensity (x_3_) influence pigment biosynthesis in *Dunaliella tertiolecta*. These models incorporate linear, quadratic, and interaction terms, enabling accurate prediction of pigment yields. For chlorophyll a (Equation (1)), the intercept value of 6.67 mg/g dw represents baseline production at central levels of the factors. Negative linear coefficients for nitrogen (−1.89) and salinity (−0.15) indicate that increasing these factors individually reduces chlorophyll a, while light intensity has a slight positive effect (0.02). A significant quadratic nitrogen term ((x_1_ − 0.55)^2^ × 13.41) suggests an optimal nitrogen level for maximum pigment yield. Interaction terms reveal synergistic effects between nitrogen and salinity ((x_1_ − 0.55)(x_2_ − 35) × 0.78) and antagonistic effects between nitrogen and light ((x_1_ − 0.55)(x_3_ − 10) × −0.97), underscoring the need to balance nutrients and stressors for optimal pigment production.

The predictive models for chlorophyll b (Equation (2)) and chlorophyll c (Equation (3)) reveal distinct regulatory patterns, highlighting the complexity of pigment synthesis in response to cultivation conditions. For chlorophyll b, the intercept (2.55 mg/g dw) reflects baseline production, with nitrogen showing a positive linear effect (0.22), unlike its negative effect on chlorophyll a—suggesting that moderate nitrogen levels may favor chlorophyll b synthesis. A significant quadratic nitrogen term ((x_1_ − 0.55)^2^ × 7.32) and interactions with salinity and light indicate nonlinear, cross-regulatory responses. For chlorophyll c, the intercept is 1.90 mg/g dw, with nitrogen showing a negative linear effect (−0.43), similar to chlorophyll a. However, quadratic and interaction effects—particularly nitrogen–salinity ((x_1_ − 0.55)(x_2_ − 35) × 0.22) and nitrogen–light ((x_1_ − 0.55)(x_3_ − 10) × −0.25)—play key roles, suggesting pigment production depends on both individual and combined factor effects. These models provide a valuable framework for optimizing pigment yields in microalgae-based bioprocesses.

### 2.3. Three-Dimensional Response Surface Modeling of Chlorophyll a, b, and c Production in Dunaliella tertiolecta

**Figure 4:**
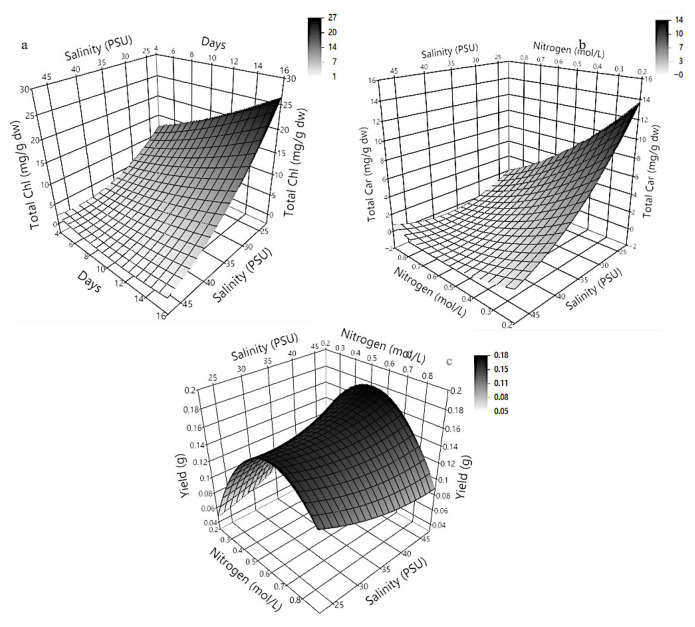
Tridimensional response surface representing chlorophyll a (**a**), chlorophyll b (**b**) and chlorophyll c (**c**) production (of the microalgae *Dunaliella tertiolecta* in function of salinity variation (PSU) and age of culture (harvest day) with nitrogen concentration (mol·L^−^^1^ of NaNO_3_) as the central point.

The three-dimensional response surface plots presented in Figure 4 illustrate the predicted behavior of chlorophyll a, b, and c production in *Dunaliella tertiolecta* as a function of salinity (PSU) and culture age (days), with nitrogen concentration maintained at the central point of the experimental design. These models offer a comprehensive visualization of the curvature effects and interactive influence of the two independent variables on pigment biosynthesis, providing key insights into the physiological responses of the microalga under varying environmental conditions.

The response surface for chlorophyll a (Figure 4a) clearly shows that maximum pigment accumulation occurs at lower salinity levels combined with an extended cultivation period. The plot exhibits a convex curvature, where chlorophyll a production significantly increases with longer culture durations while remaining inversely correlated with salinity. This suggests that prolonged exposure to mild saline conditions facilitates the accumulation of chlorophyll a, likely due to the activation of adaptive stress responses that promote chlorophyll biosynthesis as part of the photosynthetic machinery reinforcement. At higher salinity levels, pigment accumulation decreases, probably due to osmotic stress, which can impair chloroplast integrity and photosystem stability, reducing the efficiency of chlorophyll synthesis [38].

Similarly, the surface response for chlorophyll b (Figure 4b) demonstrates a comparable trend, where the pigment yield improves under lower salinity and extended cultivation time. However, the curvature for chlorophyll b appears slightly less steep compared to chlorophyll a, indicating that chlorophyll b production is also sensitive to environmental stress but may exhibit different regulatory dynamics or stability under saline stress. This differential behavior could be attributed to the specific roles of chlorophyll b within the light-harvesting complexes (LHC II), which may allow partial maintenance of photosynthetic function even under moderate salinity. In the case of chlorophyll c (Figure 4c), the response surface shows a similar dependence on salinity and culture age, with the highest production observed at the lowest salinity and longest cultivation period tested. The plot highlights a gradual and continuous increase in chlorophyll c accumulation as salinity decreases and culture age increases, suggesting a sustained biosynthetic capacity of the algal cells for this pigment under prolonged non-stressful conditions. Chlorophyll c, being part of the peripheral antenna complexes in some algal groups, might play a photoprotective role or be involved in fine-tuning energy transfer under stress adaptation mechanisms.

At the molecular level, these findings can be attributed to metabolic shifts triggered by nitrogen limitation and salinity stress, which modulate the transcription of chlorophyll biosynthetic enzymes like glutamyl-tRNA reductase and protochlorophyllide oxidoreductase. Prolonged culture under suboptimal salinity may redirect carbon fluxes toward pigment biosynthesis rather than biomass, increasing chlorophyll content per cell. These adaptive responses align with previous reports showing that environmental stress enhances pigment composition to optimize photosynthesis and protect against oxidative damage. The generated response surfaces confirm the significant roles of salinity and culture age in chlorophyll production and serve as predictive tools for optimizing cultivation. Adjusting these parameters enables maximum pigment yields in *D. tertiolecta* cultures, offering a strategic approach for nutraceutical or biotechnological applications. Overall, these results underscore the need to integrate environmental stress management with nutrient regulation to enhance pigment productivity in microalgal bioprocesses [25,39,40].

### 2.4. Optimizing Key Factors for the Yield and Biosynthesis of Total Chlorophyll and Total Carotenoids

The Central Composite Design (CCD) matrix presented in Table 2 provides a comprehensive evaluation of the effects of nitrogen concentration (X_1_), salinity (X_2_), and culture age (X_3_) on total chlorophyll (T-Chl), total carotenoid (T-Car) content, and overall biomass yield in *Dunaliella tertiolecta*. The experimental results reveal significant variability in pigment production and yield across the 15 experimental runs, confirming the critical influence of the tested factors and their interactions. The highest total chlorophyll concentration (23 mg/g dw) and carotenoid content (13.26 mg/g dw) were achieved in Run 10, under conditions of low nitrogen (0.22 mol·L^−1^), low salinity (25 PSU), and extended culture age (15 days). This result aligns with the previously discussed stress-induced enhancement of pigment biosynthesis, where nitrogen limitation and lower salinity trigger adaptive responses, redirecting metabolic fluxes toward secondary metabolite production rather than biomass accumulation.

Conversely, the lowest pigment concentrations were observed under high nitrogen and elevated salinity at early culture stages (e.g., Runs 2 and 4), where chlorophyll and carotenoid levels were reduced, indicating a prioritization of cell proliferation over pigment synthesis. Notably, higher pigment content did not always correlate with biomass yield, suggesting that pigment accumulation is more linked to stress adaptation than growth. For instance, Run 6 achieved the highest yield (0.166) but showed relatively low pigment levels, reflecting a trade-off between biomass formation and pigment biosynthesis. The predictive responses from the statistical model closely matched experimental values, validating the robustness of the quadratic equations and the CCD approach. This consistency, especially under high pigment-yielding conditions, confirms the model’s effectiveness in capturing the complex interactions among nitrogen, salinity, culture age, and pigment production. These insights are key for optimizing *D. tertiolecta* cultivation strategies aimed at enhancing pigment output for nutraceutical, pharmaceutical, and biotechnological applications [33,40,41].

The statistical evaluation offers key insights into the effects and interactions influencing total chlorophyll, carotenoids, and biomass yield in *Dunaliella tertiolecta* under the CCD framework. Nitrogen concentration (X_1_), salinity (X_2_), and culture age (X_3_) were assessed through F values and significance levels (Prob > F). For total chlorophyll, salinity (X_2_) was the most significant factor (F = 17.4779, Prob > F = 0.0058 *), emphasizing the impact of osmotic stress on pigment biosynthesis. Notably, the salinity–light (X_2_ × X_3_; F = 11.7467, Prob > F = 0.0140 **) and nitrogen–light (X_1_ × X_3_; F = 16.7579, Prob > F = 0.0064 *) interactions were significant, showing that pigment accumulation depends on combined environmental and nutritional conditions. The nitrogen–salinity interaction (X_1_ × X_2_; F = 33.1315, Prob > F = 0.0012 *) also had a strong influence, suggesting that salinity can modulate nitrogen-driven chlorophyll synthesis. These findings highlight the need to optimize multiple factors to maximize pigment yield simultaneously.

Regarding total carotenoid production, salinity (X_2_) once again demonstrated a significant linear effect (F = 6.1783, Prob > F = 0.0474 **), while the nitrogen–salinity interaction (X_1_ × X_2_) showed a very strong influence (F = 10.9321, Prob > F = 0.0163 **), reinforcing the evidence of interactive regulation between these two factors on pigment biosynthesis pathways. The nitrogen–culture age interaction (X_1_ × X_3_) was also statistically significant (F = 6.4316, Prob > F = 0.0443 **), suggesting that the duration of cultivation plays a vital role in maximizing carotenoid production under specific nitrogen conditions. These results align with the known physiological mechanisms in microalgae where nitrogen limitation, combined with osmotic modulation and sufficient cultivation time, enhances the flux toward secondary metabolite pathways, particularly carotenoids, which function as antioxidants and photoprotective molecules.

For biomass yield, culture age (X_3_) was the most influential factor (F = 55.0668, Prob > F = 0.0003 *), indicating that longer cultivation significantly enhances biomass accumulation, consistent with typical microalgal growth patterns. The nitrogen–salinity interaction (X_1_ × X_2_) showed the highest F value among interactions (F = 25.1315, Prob > F = 0.0024*), highlighting the critical role of balancing nutrients and salinity for optimal productivity. A significant quadratic effect of nitrogen (X_1_ × X_1_; F = 73.4496, Prob > F = 0.0001 *) revealed a nonlinear relationship, where both low and high nitrogen levels can impair growth. Additionally, the nitrogen–culture age interaction (X_1_ × X_3_; F = 11.7652, Prob > F = 0.0140 **) and the quadratic term for culture age (X_3_ × X_3_; F = 9.4601, Prob > F = 0.0218 **) were significant, suggesting that biomass yield depends on a precise combination of nutrient input and cultivation time to avoid growth limitations or inefficiencies.

Collectively, these findings provide robust statistical evidence that pigment production and biomass yield in *D. tertiolecta* are modulated not only by individual cultivation parameters but also by their interactions, supporting the need for an integrative optimization strategy. These results confirm the effectiveness of the CCD approach and response surface methodology as predictive tools for optimizing pigment production and biomass yield in microalgal cultivation, enabling the strategic design of efficient bioprocesses [33]. To support these findings and generate predictive models, second-order polynomial equations were developed using JMP V11. This enabled precise estimation of response variables and visualization of factor interactions via response surface methodology. The predicted ranges were 1.26–23 mg/g dw for total chlorophyll (Equation (4)), 0.11–13.26 mg/g dw for carotenoids (Equation (5)), and 0.018–0.133 g for yield (Equation (6)).

12.7978965517241 + (−2.0909090909091 × x_1_) + (−0.2778 × x_2_) + (0.0508 × x_3_) + ((x_1_ − 0.55) × ((x_1_ − 0.55) × 33.0546847788227)) + ((x_1_ − 0.55) × ((x_2_ − 35) × 1.29583333333333)) + ((x_2_ − 35) × ((x_2_ − 35) × 0.02449655172414)) + ((x_1_ − 0.55) × ((x_3_ − 10) × −1.8431818181818)) + ((x_2_ − 35) × ((x_3_ − 10) × −0.050925)) + ((x_3_ − 10) × ((x_3_ − 10) × 0.02918620689655)) (4)

5.65984482758621 + (−3.8272727272727 × x_1_) + (−0.1407 × x_2_) + (0.144 × x3) + ((x_1_ − 0.55) × ((x_1_ − 0.55) × 10.09626040974)) + ((x_1_ − 0.55) × ((x_2_ − 35) × 0.63409090909091)) + ((x_2_ − 35) × ((x_2_ − 35) × 0.01199482758621)) + ((x_1_ − 0.55) × ((x_3_ − 10) × −0.9727272727273)) + ((x_2_ − 35) × ((x_3_ − 10) × −0.02635)) + ((x_3_ − 10) × ((x_3_ − 10) × 0.02377931034483)) (5)

0.01347011494253 + (−0.010303030303 × x_1_) + (0.00122 × x_2_) + (0.00566 × x_3_) + ((x_1_ − 0.55) × ((x_1_ − 0.55) × −0.584528672303)) + ((x_1_ − 0.55) × ((x_2_ − 35) × −0.0064772727273)) + ((x_2_ − 35) × ((x_2_ − 35) × 0.00006344827586)) + ((x_1_ − 0.55) × ((x_3_ − 10) × 0.00886363636364)) + ((x_2_ − 35) × ((x_3_ − 10) × −0.0001125)) + ((x_3_ − 10) × ((x_3_ − 10) × 0.00091379310345)) (6)

The second-order polynomial equations for total chlorophyll (Equation (4)), total carotenoids (Equation (5)), and biomass yield (Equation (6)) offer a solid framework for predicting these responses under different cultivation conditions. Each model includes linear, quadratic, and interaction terms, capturing the complex effects of nitrogen concentration (x_1_), salinity (x_2_), and culture age (x_3_). For total chlorophyll (Equation (4)), the intercept (12.80 mg/g dw) represents baseline production under central conditions. Negative linear coefficients for nitrogen (−2.09) and salinity (−0.28) suggest that higher values reduce chlorophyll synthesis, while the positive effect of culture age (0.05) indicates that longer cultivation favors pigment accumulation. The significant nitrogen quadratic term ((x_1_ − 0.55)^2^ × 33.05) reveals a strong curvature, pointing to an optimal nitrogen level. Interaction effects—particularly between nitrogen and salinity (1.29), and nitrogen and culture age (−1.84)—highlight the importance of combined optimization, as these variables can act synergistically or antagonistically in modulating chlorophyll biosynthesis.

Equation (5) for total carotenoids follows a similar structure to chlorophyll but reveals distinct factor influences. The intercept (5.66 mg/g dw) sets the baseline, with nitrogen showing a strong negative linear effect (−3.83) and culture age a moderate positive effect (0.14), indicating that nitrogen limitation and prolonged cultivation promote carotenoid accumulation, likely as a stress response. The significant quadratic nitrogen term ((x_1_ − 0.55)^2^ × 10.10) confirms a nonlinear behavior with an optimal nitrogen level. Interactions—particularly nitrogen–salinity (0.63) and nitrogen–culture age (−0.97)—also influence carotenoid biosynthesis. For biomass yield (Equation (6)), the intercept is 0.0135 g. Nitrogen negatively affects yield (−0.0103), while salinity (0.00122) and culture age (0.00566) contribute positively. The nitrogen quadratic term ((x_1_ − 0.55)^2^ × −0.58) suggests yield decreases at extreme nitrogen levels. Although interaction effects are smaller, they confirm that yield depends on the combined influence of all factors. These models enable strategic optimization in microalgal bioprocesses.

The analysis of the Central Composite Design (CCD) and statistical modeling showed that total chlorophyll, carotenoid production, and biomass yield in *Dunaliella tertiolecta* are significantly influenced by the interactions between nitrogen concentration, salinity, and culture age. ANOVA results identified salinity and culture age as key for chlorophyll and biomass, while nitrogen, especially under limitation, strongly affected carotenoids. Significant quadratic and interaction terms confirmed the system’s nonlinear nature, indicating that optimal pigment and biomass levels require balancing all variables rather than adjusting one in isolation [33,40,41]. These findings align with known stress responses in microalgae, where nitrogen limitation and osmotic stress enhance secondary metabolite pathways as adaptive mechanisms.

### 2.5. Three-Dimensional Response Surface Modeling of Total Chlorophyll, Total Carotenoids Production and Microalgal Biomass Yield in Dunaliella tertiolecta

**Figure 5:**
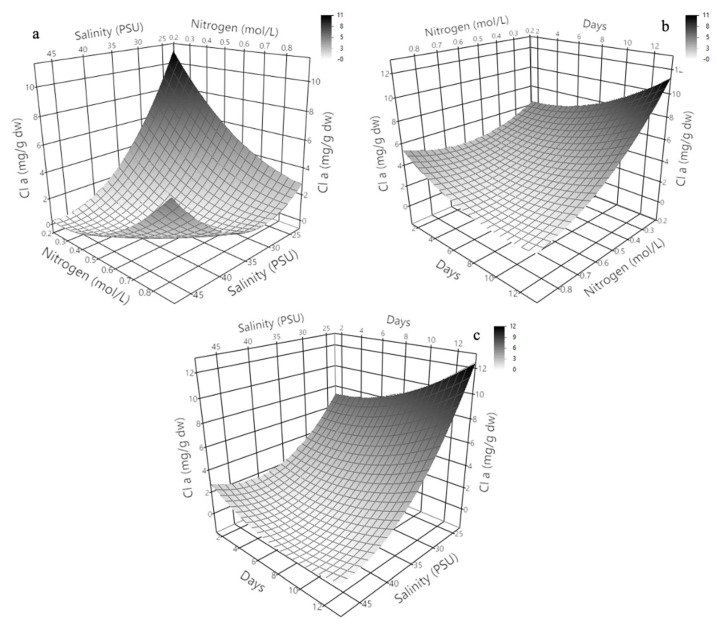
Tridimensional response surface representing total chlorophyll, carotenoids and yields production of the microalgae *Dunaliella tertiolecta* in function of nitrogen concentration (mol·L^−^^1^ of NaNO_3_) and salinity variation (PSU) on day 15 (**a**); nitrogen concentration and age of culture (days) at 25 PSU (**b**) and salinity and age of culture with 0.22 mol·L^−^^1^ of NaNO_3_ (**c**).

The three-dimensional response surface plots presented in Figure 5 illustrate the predictive modeling of total chlorophyll (Figure 5a), total carotenoids (Figure 5b), and biomass yield (Figure 5c) in *Dunaliella tertiolecta*, providing a comprehensive view of how variations influence these responses in salinity (PSU), culture age (days), and nitrogen concentration (mol·L^−1^ NaNO_3_). These plots, generated from the second-order polynomial equations derived through the Central Composite Design (CCD), effectively capture the curvature and interactive effects between the evaluated parameters, offering valuable insights into the physiological behavior and metabolic regulation of this microalga under different cultivation conditions.

The response surface analysis for total chlorophyll (Figure 5a) and total carotenoids (Figure 5b) in *Dunaliella tertiolecta* highlights the critical influence of salinity, culture age, and nitrogen concentration on pigment biosynthesis. Chlorophyll accumulation increases with extended cultivation and decreases with higher salinity, reaching maximum levels at low salinity (~25 PSU) and long culture durations (~15 days). This suggests that mild osmotic stress and prolonged growth favor chlorophyll production as part of the cell’s adaptive response, likely mediated by the upregulation of biosynthetic enzymes under nitrogen limitation and low salinity to optimize light capture. Similarly, carotenoid production follows this trend but shows greater sensitivity to nitrogen levels. Peak carotenoid accumulation occurs at low salinity and reduced nitrogen, supporting the role of nitrogen deprivation as a strong inducer of secondary carotenoid biosynthesis. The nonlinear curvature of the response surface confirms that carotenoid production declines at higher nitrogen levels, even under optimal salinity, due to a metabolic shift toward growth rather than stress-related metabolite synthesis. These findings align with known stress-response mechanisms in microalgae, where nitrogen limitation redirects metabolism toward antioxidant and photoprotective pigment production, enhancing cellular defense under unfavorable conditions.

The response surface for biomass yield (Figure 5c) reveals a distinct pattern, with maximum yield achieved at moderate nitrogen concentrations and intermediate salinity, while culture age has a lesser impact. Unlike pigment accumulation, which benefits from nitrogen limitation and low salinity, these stress conditions reduce biomass growth, highlighting a metabolic trade-off between proliferation and secondary metabolite production. The peak yield near the central nitrogen level indicates that sufficient nitrogen is essential for cell growth, whereas excessive limitation hinders biomass despite enhancing pigment synthesis. These models illustrate the complex regulation of pigment and biomass production in *D. tertiolecta*, shaped by the interplay of salinity, nitrogen, and culture duration. The findings confirm the utility of the CCD approach and response surface methodology in predicting outcomes and offer practical strategies to optimize cultivation—whether aiming to increase pigment content for nutraceutical use or biomass for bioresource applications—by adjusting environmental conditions according to specific production goals [38].

The optimization of cultivation parameters using Central Composite Design and response surface methodology identified nitrogen concentration, salinity, and culture age as key factors influencing pigment biosynthesis and biomass yield in *Dunaliella tertiolecta* [36,37,38]. Optimal pigment production—23 mg/g dw of chlorophyll and 13.26 mg/g dw of carotenoids—was achieved under low nitrogen (0.22 mol·L^−1^ NaNO_3_), low salinity (25 PSU), and extended culture (15 days), as shown in Run 10. These conditions promote a stress-induced metabolic shift toward secondary metabolite accumulation while maintaining acceptable biomass yield. In contrast, higher nitrogen levels enhanced biomass but suppressed pigment synthesis, confirming a trade-off between growth and pigment production. Thus, the selected conditions strategically favor high-value pigment output for biotechnological applications. All subsequent analyses, including pigment encapsulation into nanoliposomes and their physicochemical characterization, will use biomass from this optimized condition to ensure consistency and relevance in evaluating the developed delivery systems.

### 2.6. High-Performance Liquid Chromatography (HPLC) of Pigment-Rich Optimized Microalgae Extract

The HPLC chromatogram presented in Supplementary Material Figure S1 and Table 3 shows the pigment profile of the *Dunaliella tertiolecta* extract, obtained at a detection wavelength of 457 nm, which is characteristic of carotenoids due to their strong absorbance in the blue region of the visible spectrum. The chromatographic separation revealed seven distinct peaks, each corresponding to different pigments present within the extract. The early retention peaks (particularly peaks 1 and 2) likely represent more polar carotenoids such as all-trans violaxanthin and 13-cis lutein, which generally elute earlier due to their hydroxylated structures increasing polarity. All-trans-violaxanthin is the pigment more abundant in the *Dunaliella tertiolecta* extract. Peaks 3 may correspond to mono-epoxy carotenoid (all-trans luteoxanthin), which are commonly found in microalgal pigment profiles. Peak 4 is all-trans zeaxanthin, which, according to bibliographic references, is common in microalgae [42]. The last peaks (5–7) are characteristic of less polar carotenoids as all-trans α-carotene, all-trans β-carotene, and 9-cis-β-carotene. The latter is one of the most abundant carotenoids reported in *Dunaliella* species, particularly under nitrogen limitation or stress conditions where β-carotene accumulation is enhanced [12,42]. Similar results are obtained by some references [43,44]. Unfortunately, some peaks could not be identified, representing a quantification of 6.79%.

Structurally, β-carotene is a highly conjugated polyene hydrocarbon with no oxygen-containing functional groups, which contributes to its non-polar nature and its later elution in reverse-phase HPLC systems. Extensive π-conjugation systems facilitate strong light absorption. This behavior contrasts with other xanthophyll carotenoids (such as lutein and violaxanthin) that contain hydroxyl or epoxy groups, increasing their polarity and resulting in earlier retention times.

The presence of β-carotene in the *D. tertiolecta* extract aligns with the known metabolic pathways of this species, where the carotenoid biosynthesis involves enzymes such as phytoene synthase, lycopene cyclase, and β-carotene hydroxylase. Under stress conditions like high light intensity or nutrient deprivation, the expression of these enzymes is modulated, leading to the preferential accumulation of β-carotene as a photoprotective strategy. This molecular adaptation not only supports light harvesting and reactive oxygen species quenching but also explains the dominance of β-carotene in the pigment profile, as evidenced by one of the major peaks observed in the chromatogram [12,42,43,44].

Although all-trans violaxanthin was the major compound in the *D. tertiolecta* extract, β-carotene has some advantages over this compound. β-carotene is a precursor of vitamin A, essential for vision, immunity, and growth. Violaxanthin does not have significant provitamin A activity. Furthermore, β-carotene is generally more stable to heat and light compared to violaxanthin, which contains labile epoxides. Furthermore, β-carotene is widely approved as an additive (E160a) by the FDA (Food and Drug Administration) and EFSA (European Food Safety Authority); it is used in beverages, snacks, and supplements. Violaxanthin is not as widely regulated. Although both have antioxidant activity, β-carotene effectively neutralizes lipophilic free radicals, making it more versatile in biological systems [6]. Therefore, β-carotene is superior to violaxanthin in many contexts, highlighting its provitamin A activity, stability, bioavailability, and industrial and nutritional applications. Consequently, the interest in its placement in nanoliposomes improves its stability against oxidation and light, increases its bioavailability and intestinal absorption, and allows for controlled and targeted release in nutraceutical applications [15]. Furthermore, this technology protects its antioxidant activity and enhances its physiological effects [11,20,22,45].

### 2.7. Nano-Sized Liposomes via the Particle Dispersion Method

**Figure 6:**
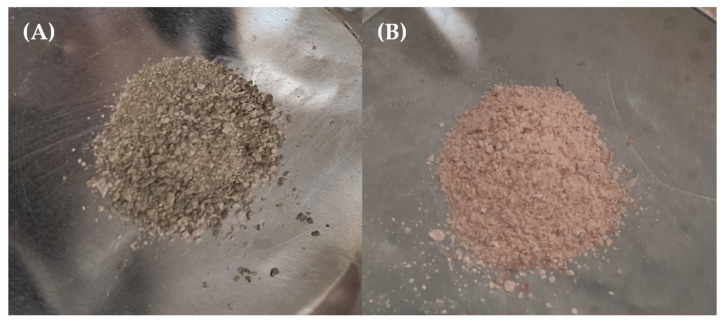
Microalgae extract-loaded nanoliposome (**A**) and β-carotene-loaded nanoliposome (**B**).

The extract from the optimized microalgal biomass (0.22 mol·L^−1^ NaNO_3_; 25 PSU; 15 days) and the β-carotene (food-grade by Sigma-Aldrich Co.) were encapsulated in nano-liposomal vehicles composed of phosphatidylcholine and cholesterol using the particle dispersion method. A final volume of 100 mL of each nano-liposomal vehicle, loaded and suspended in saline solution, was obtained and stored in freezing conditions and darkness until the lyophilization process. Once lyophilized, the yield was 245 mg of nano-liposomes loaded with microalgal extract (Figure 6A) and 260 mg of nano-liposomes loaded with β-carotene (Figure 6B).

### 2.8. Encapsulation Efficiency

The evaluation of encapsulation efficiency highlighted clear differences between nanoliposomes loaded with Dunaliella tertiolecta extract and those formulated with pure β-carotene. Nanoliposomes containing the microalgal extract showed an efficiency of 63.78 ± 2.24%, while those with β-carotene alone reached a markedly higher value of 94.67 ± 3.56%. This contrast can be explained by the heterogeneous composition of the extract, which comprises a blend of seven carotenoids, including all-trans violaxanthin, 13-cis lutein, all-trans luteoxanthin, all-trans zeaxanthin, all-trans α-carotene, all-trans β-carotene, and 9-cis-β-carotene, as identified through HPLC [12,42,43,44]. The diversity in polarity, isomeric configuration, and unsaturation among these compounds may generate competition for integration into the liposomal bilayer, reducing their overall encapsulation. In particular, xanthophylls such as violaxanthin and luteoxanthin possess polar groups that can interfere with stable incorporation into the hydrophobic lipid core, promoting their partial exclusion from the vesicles or their localization at the bilayer surface rather than full entrapment [45,46].

At the interactional molecular level, β-carotene’s superior encapsulation can be explained by its highly hydrophobic, planar, and symmetric polyene structure, which facilitates its stable incorporation into the hydrophobic region of the phospholipid bilayer. The extended system of conjugated double bonds allows for favorable van der Waals interactions and π-π stacking with the acyl chains of the phospholipids, contributing to its thermodynamic compatibility with the lipid environment. In contrast, the carotenoid mixture in *D. tertiolecta* extract introduces steric hindrance, varying degrees of polarity, and cis-isomerization that may reduce the efficiency of entrapment. Additionally, possible competitive interactions between carotenoids in the extract and the lipid headgroups or aqueous interface may further limit encapsulation. These molecular dynamics highlight the importance of compound-lipid compatibility and the physicochemical nature of bioactives when designing nanocarrier systems for complex natural extracts [47].

### 2.9. Determination of Particle Size and Zeta Potential Stability

**Figure 7:**
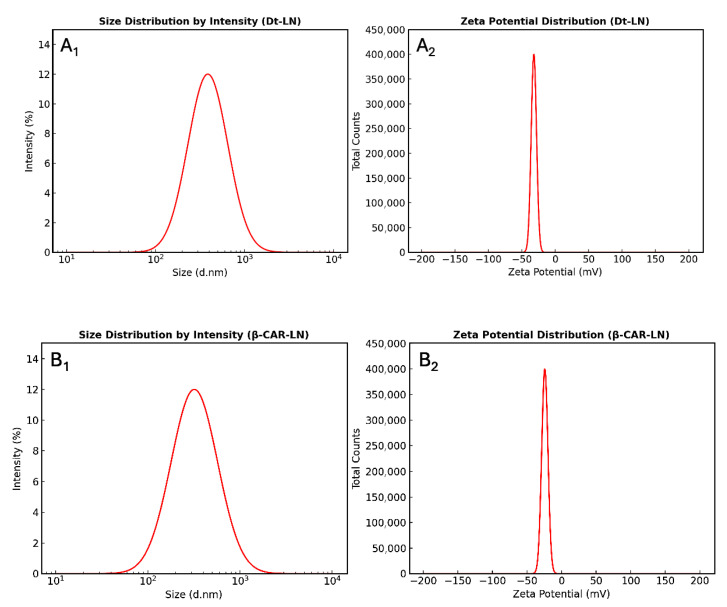
Figure depicting the average particle size (**A_1_**,**B_1_**) and zeta potential (**A_2_**,**B_2_**) of Dt-LN, β-CAR-LN.

The results obtained revealed clear differences between the nanoliposomal systems in terms of homogeneity and colloidal stability (Table 4). The microalgae extract-loaded nanoliposomes, with an encapsulation efficiency (EE) of 63.78 ± 2.24% and an average particle size of 387 ± 21.32 nm, exhibited a low polydispersity index (PDI, 0.26 ± 0.057), indicating a relatively uniform particle distribution with a single dominant peak. This behavior suggests that the lipid bilayer was efficiently organized to retain the bioactive compound, minimizing the formation of subpopulations or aggregates. Moreover, the zeta potential value (–32 ± 0.97 mV) places the system within the electrostatic stability range, preventing vesicle aggregation or fusion (Figure 7(A_1_,A_2_)). Taken together, these parameters explain the higher encapsulation efficiency observed, as a sufficiently negative surface charge favors electrostatic repulsion among nanoliposomes, thereby preserving the entrapped bioactive compound.

In contrast, β-carotene-loaded liposomes (EE = 94.67 ± 3.56%, particle size 320 ± 12.92 nm, PDI = 0.33 ± 0.037, ζ = –24.23 ± 1.23 mV) showed greater heterogeneity and moderate colloidal stability (Figure 7(B_1_,B_2_)). The higher PDI suggests the presence of secondary particle populations, while the lower zeta potential indicates a weaker electrostatic barrier against aggregation, favoring premature release of the bioactive compound. Overall, although both formulations fall within the acceptable range of nanometric size and stability, the microalgae extract nanoliposomes appear more robust and efficient, positioning them as a promising alternative for nutraceutical or functional food applications.

Our findings contrast with those reported by Pan et al. [18], who developed astaxanthin-loaded nanoliposomes with significantly smaller particle sizes (60–80 nm) and a notably higher EE (97%), demonstrating a more efficient and homogeneous system. The discrepancy could be attributed to methodological differences, since Pan et al. [18] employed high-energy ultrasound, which effectively reduced particle size and improved size distribution uniformity. Despite our nanoliposomes exhibiting larger dimensions, the PDI and zeta potential values (–32 mV) obtained in this study indicate adequate colloidal stability, supporting their applicability in functional food matrices. Similarly, β-carotene liposomes in our study showed moderate stability and reduced encapsulation, partially aligning with the observations of Jiao et al. [48]. These authors demonstrated that surface modification with poly-L-lysine (PLL) enhanced EE to 92.49% and reduced PDI from 0.451 to 0.347, highlighting the critical role of liposomal surface properties in encapsulation efficiency and stability. In contrast, the absence of surface decoration in our system resulted in lower EE, underscoring the importance of liposomal engineering strategies to optimize the bioavailability of lipophilic compounds such as β-carotene.

Taken together, the comparison with Pan et al. [18] and Jiao et al. [48] provides valuable context for our findings. While Pan et al. [18] emphasize the importance of particle size reduction and homogeneity to maximize EE, Jiao et al. [48] demonstrate the role of surface functionalization in improving stability and encapsulation. Our results occupy an intermediate position: the microalgae extract system demonstrated greater homogeneity and stability, whereas β-carotene liposomes presented limitations linked to surface charge and polydispersity. These differences reinforce the notion that both processing conditions and structural modifications are critical determinants of nanoliposome performance, opening avenues to explore high-energy ultrasound and polymeric surface decoration in future work.

### 2.10. Centrifugal Stability Measurement

The centrifugal stability measurement revealed a significant difference between the two nanoliposomal formulations: β-CAR-LN exhibited a high stability of 87.75 ± 2.38%, while Dt-LN showed a markedly lower stability of 38.76 ± 3.64%. This disparity reflects the intrinsic chemical and structural differences between the encapsulated compounds and their molecular interactions with the liposomal bilayer. β-carotene, being a highly hydrophobic molecule with a linear, symmetric polyene chain, exhibits excellent compatibility with the hydrophobic core of the phospholipid bilayer. Its uniform structure facilitates deep insertion and strong van der Waals and π–π stacking interactions with the acyl chains of the phospholipids, leading to a compact, well-integrated liposomal structure with minimal leakage during centrifugation. In contrast, the *Dunaliella tertiolecta* extract (Dt-LN) contains a heterogeneous mixture of seven structurally diverse carotenoids, including polar xanthophylls (e.g., violaxanthin and luteoxanthin), and both cis and trans isomers. These molecules differ in polarity, isomeric configuration, and functional groups, which can disrupt the lipid packing of the bilayer, reducing vesicle integrity and promoting phase separation or surface localization of some compounds. The lower centrifugal stability of Dt-LN correlates with its low encapsulation efficiency (43.45%), indicating that a significant portion of the extract remains weakly associated with the lipid interface or exists outside the vesicles. Structurally, the presence of bulky, oxygenated carotenoids may create steric hindrance and limit their incorporation into the hydrophobic bilayer, resulting in poor physical stability under centrifugal stress. This compromised stability could lead to premature release or degradation of bioactives, thereby reducing the functional efficacy of the nanocarrier system. Conversely, the high stability of β-CAR-LN aligns with its high encapsulation efficiency (94.67%), confirming the importance of molecular compatibility in ensuring both efficient loading and structural robustness of nanoliposomes. These results highlight the necessity of tailoring the lipid composition and encapsulation strategy based on the physicochemical nature of the encapsulated compounds to achieve optimal performance in delivery systems [49].

### 2.11. In Vitro Release

**Figure 8:**
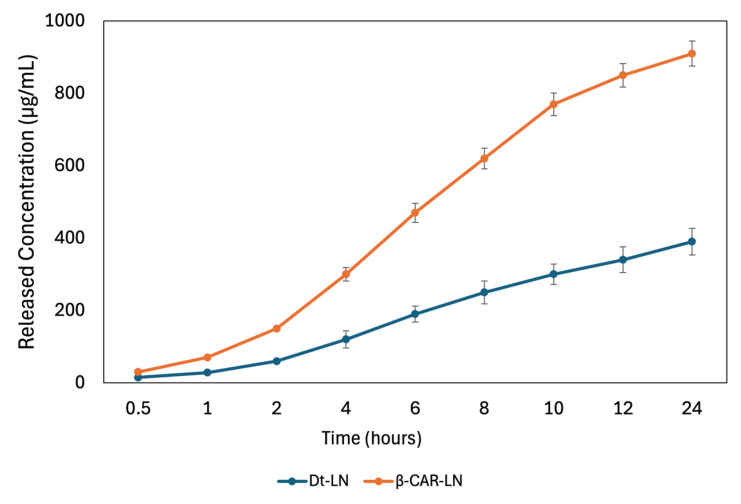
In vitro release profiles of *Dunaliella tertiolecta* extract (Dt-LN) and pure β-carotene (β-CAR-LN) from nanoliposomes over a 24-h period. The released concentration (µg/mL) was measured at predetermined time intervals.

The in vitro release kinetics of both the *Dunaliella tertiolecta* extract and pure β-carotene from nanoliposomes revealed distinct behaviors associated with their molecular structure and compatibility with the lipid bilayer (Figure 8). The release profile of the *D. tertiolecta* extract showed a gradual increase in concentration over time, reaching a cumulative release of approximately 390 µg/mL after 24 h. In contrast, β-carotene exhibited a more sustained and higher release, reaching up to 891 µg/mL under the same conditions. These results correlate with their respective encapsulation efficiencies, 43.45% for the *D. tertiolecta* extract and 94.67% for β-carotene, and confirm that compounds with higher affinity to the liposomal core are released in a more controlled and efficient manner. Structurally, β-carotene is a highly hydrophobic molecule with a linear, symmetric polyene chain, which allows deep insertion into the hydrophobic region of the liposomal membrane. This results in strong van der Waals and π–π stacking interactions with phospholipid acyl chains, stabilizing the encapsulation and facilitating a gradual diffusion-based release [49].

**Figure 9:**
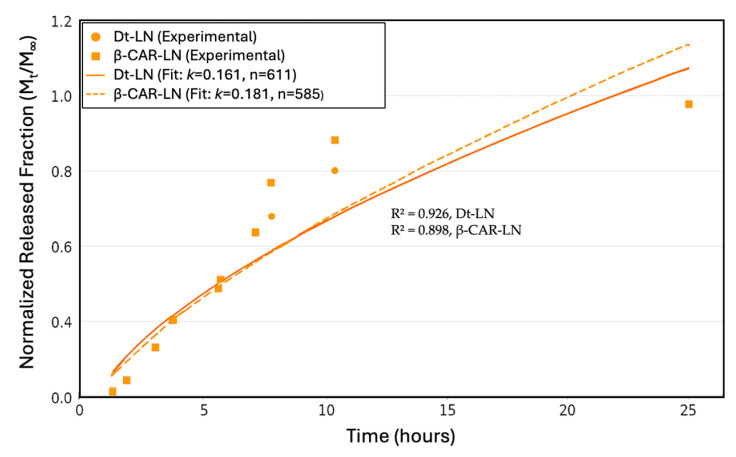
Korsmeyer–Peppas kinetic model fitting describing the in vitro release of *Dunaliella tertiolecta* extract and β-carotene from nanoliposomes. The normalized released fraction (Mt/M∞) was plotted as a function of time, showing anomalous release kinetics for both systems. Experimental data fitted the model with high correlation, indicating a combined mechanism of diffusion and lipid matrix relaxation. M_t_ = Amount of compound released at time t; M_∞_: total amount released at equilibrium (∞); *k* = kinetic constant (related to the release rate); *n* = release exponent (indicates the transport mechanism). R^2^ = Represents the goodness of fit (coefficient of determination).

The Korsmeyer–Peppas model was applied to describe the release mechanisms, yielding an exponent *n* = 0.611 for the *D. tertiolecta* extract and *n* = 0.585 for β-carotene, with corresponding release rate constants *k* of 0.161 and 0.181, respectively (Figure 9). These values suggest that both systems follow a non-Fickian (anomalous) transport mechanism, where a combination of diffusion governs the release through the lipid matrix and structural relaxation or erosion of the liposomal bilayer. The model’s fit was strong for both formulations, with R^2^ values of 0.926 for the *D. tertiolecta* extract and 0.898 for β-carotene, indicating that the kinetic model adequately captures the release behavior. The slightly higher R^2^ for the extract implies a better predictability of the model in more complex mixtures, despite its lower encapsulation and stability. This may be due to the presence of polar carotenoids (such as violaxanthin and luteoxanthin) that preferentially localize at the lipid–aqueous interface and are released earlier, while less polar carotenoids like β-carotene remain embedded and are released more slowly. Collectively, these findings emphasize the critical role of compound–lipid compatibility in determining both the stability and release dynamics of nanoliposomal delivery systems. This type of analysis is crucial for optimizing controlled-release formulations in nutraceutical or pharmaceutical applications [50].

### 2.12. Sustained-Release Kinetics and Antioxidant Activities of Loaded Nanoliposomes

**Figure 10:**
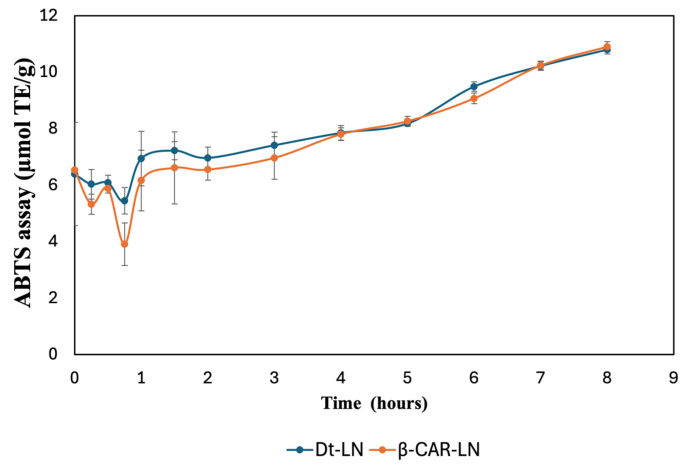
Sustained-release kinetics of the nano-liposomes loaded with microalgal extract and β-carotene on ABTS•^+^.

**Figure 11:**
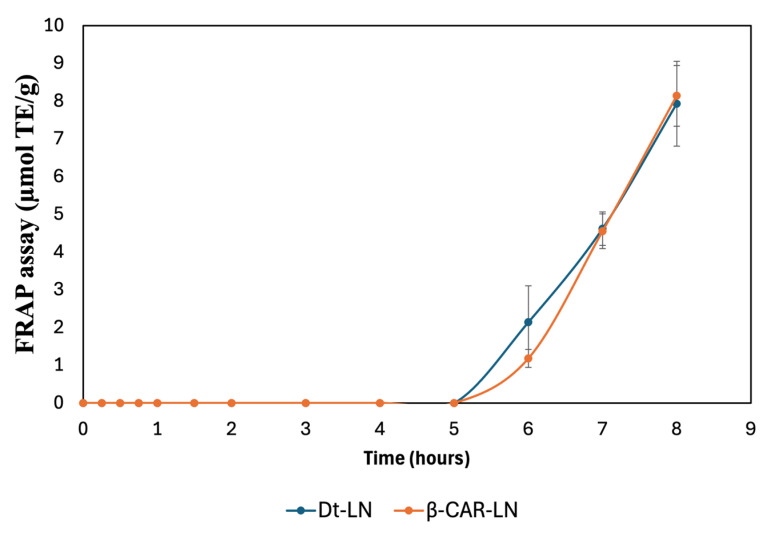
Sustained-release kinetics of the nano-liposomes loaded with microalgal extract and β-carotene on FRAP.

The ABTS•^+^ and FRAP assays were used to evaluate the antioxidant activity of the pigment-loaded nanoliposomes. Prior to the assays, 20 mg of the lyophilized samples was resuspended in 10 mL of physiological solution (0.9% sodium chloride) for each test. Absorbance measurements were taken at 0, 15, 30, and 45 min, as well as from 1 to 8 h, as shown in Figure 10 and Figure 11.

### 2.13. ABTS Assay

The results shown in Figure 10 illustrate the sustained-release kinetics and antioxidant activity of nanoliposomes loaded with the optimized microalgal extract (Dt-LN) and β-carotene (β-CAR-LN), evaluated by the ABTS•^+^ radical scavenging assay and expressed in µmol TE/g over a period of 8 h. Both nanoformulations demonstrated a controlled and gradual release profile, with a progressive increase in antioxidant activity throughout the evaluated time, suggesting effective encapsulation and sustained delivery of bioactive compounds. Initially, within the first hour, both systems exhibited a slight decrease in ABTS•^+^ inhibition, likely due to the stabilization phase of the nanoliposomes in the release medium, followed by a steady recovery and gradual increase in antioxidant potential. This initial dip may also reflect a rapid release of surface-associated compounds before the controlled release from the nanoliposomal core becomes dominant [51]. The highest antioxidant activities were recorded after 8 h of exposure to the physiological solution, yielding values of 10.79 ± 0.15 and 10.90 ± 0.18 µmol TE/g for nanoliposomes loaded with *Dunaliella tertiolecta* extract and β-carotene, respectively. A comparable trend was observed in the FRAP assay, with values of 7.93 ± 1.12 and 8.14 ± 0.81 µmol TE/g for nanoliposomes encapsulated with *D. tertiolecta* extract and β-carotene, respectively.

The Dt-LN formulation consistently showed slightly higher antioxidant activity than β-CAR-LN, especially during the early to intermediate release phases (1–6 h). This suggests that the microalgal extract, containing a diverse mix of chlorophylls, carotenoids, and other antioxidants, may exert synergistic effects that enhance radical scavenging beyond β-carotene alone [46,47,49]. The structural diversity of both polar and non-polar compounds likely contributes to broader antioxidant action. The sustained release observed is linked to the nanoliposome’s bilayer structure, which gradually releases encapsulated compounds through a semipermeable phospholipid matrix. Hydrophobic molecules like carotenoids and chlorophylls diffuse slowly through this lipid barrier. Additionally, interactions between bioactives and the lipid matrix influence release rates, with β-carotene’s stronger affinity for the hydrophobic core leading to a slightly slower release than the more complex Dt-LN mixture [50,52,53,54].

In the later stages of the kinetic profile (after 6 h), both Dt-LN and β-CAR-LN formulations reached similar antioxidant activity levels, indicating that most encapsulated compounds had been released, allowing full interaction with ABTS•^+^ radicals. This plateau confirms the nanoliposomal system’s effectiveness in sustaining antioxidant release over time—an important feature for applications in functional foods, nutraceuticals, and therapeutics. The overall results validate nanoliposomal encapsulation as a reliable strategy for prolonged delivery of antioxidant compounds from both microalgal extract and β-carotene. Notably, the superior early performance of Dt-LN underscores the benefits of complex natural extracts, which offer synergistic interactions and a broader antioxidant spectrum compared to isolated compounds. This sustained-release behavior may enhance oxidative protection in biological systems, increasing the functional and bioactive potential of the encapsulated compounds [51,55,56].

### 2.14. FRAP Assay

The results presented in Figure 11 depict the sustained-release kinetics and ferric reducing antioxidant power (FRAP) activity of nanoliposomes loaded with the optimized microalgal extract (Dt-LN) and β-carotene (β-CAR-LN). The FRAP assay specifically measures the electron-donating capacity of the released bioactive compounds by assessing their ability to reduce Fe^3+^ (ferric ion) to Fe^2+^ (ferrous ion), reflecting their potential as reducing agents and antioxidants.

The release kinetics observed in this assay clearly demonstrate a prolonged and controlled release behavior for both nanoformulations, with the antioxidant activity initiating after a lag phase and progressively increasing over time, reaching its maximum after 8 h. This gradual increase indicates that the encapsulated bioactive compounds are not released immediately but rather follow a controlled release mechanism, governed by the diffusion of the compounds through the nanoliposomal bilayer and their interaction with the lipid matrix.

The kinetic profiles of both Dt-LN and β-CAR-LN formulations show negligible ferric reducing activity during the initial 5 h of incubation, suggesting effective retention of the active compounds within the nanoliposomal system and minimal early leakage. This delayed onset is characteristic of sustained-release systems, where the encapsulated compounds diffuse slowly from the lipid core to the external medium, likely following a Fickian diffusion-controlled mechanism. The subsequent sharp increase in FRAP values after 5 h reflects the progressive release and accumulation of the bioactive molecules into the medium, where they become available to participate in the electron transfer reactions required for Fe^3+^ reduction [51,56].

At the molecular level, the FRAP assay operates through a single electron transfer (SET) mechanism, wherein antioxidants act as electron donors to reduce the ferric-tripyridyltriazine (Fe^3+^-TPTZ) complex to its ferrous form (Fe^2+^-TPTZ), which is quantified spectrophotometrically. The observed antioxidant activity, therefore, directly correlates with the presence and release rate of effective electron-donating molecules from the nanoliposomal systems. In this context, carotenoids, chlorophyll derivatives, and other antioxidant metabolites present in the microalgal extract or β-carotene are responsible for donating electrons via SET to drive the reduction process. The slow, sustained increase in FRAP activity indicates that the integrity of the nanoliposome structure effectively modulates the release, preventing the immediate availability of these reducing agents and promoting a gradual delivery instead [57].

Interestingly, although both formulations exhibited similar release patterns, the Dt-LN system showed a slightly earlier onset of FRAP activity compared to β-CAR-LN, which could be attributed to the complex composition of the microalgal extract, containing both hydrophilic and lipophilic compounds with varying affinities to the lipid bilayer. This heterogeneous mixture may facilitate a broader range of interactions with the lipid matrix, resulting in a more gradual but sustained release of the electron-donating molecules. In contrast, the β-carotene-loaded liposomes, containing a single hydrophobic molecule, may exhibit a slower diffusion process through the liposomal membrane due to the strong interaction between β-carotene and the hydrophobic lipid core.

These findings confirm that nanoliposomal encapsulation of microalgal extract and β-carotene enables controlled, sustained antioxidant release with prolonged bioactivity. The FRAP assay (SET mechanism) highlights functional release performance, supporting their potential in nutraceutical, food, or biomedical applications. This sustained-release behavior may improve oxidative balance, reduce dosing frequency, and enhance bioactive compound stability and bioavailability. The sustained-release behavior and antioxidant activity of Dt-LN and β-CAR-LN, assessed via ABTS and FRAP assays, revealed a controlled, prolonged release of encapsulated bioactives. The ABTS assay, sensitive to both hydrogen atom transfer (HAT) and single electron transfer (SET) mechanisms, showed early and continuous antioxidant activity over 8 h, indicating effective release of both surface-bound and encapsulated compounds. In contrast, the FRAP assay, which detects only SET-based activity, displayed a delayed response with a clear lag phase in the first 5 h, followed by a marked increase in reducing power. This suggests that ABTS captures early scavenging by a broader range of antioxidants, while FRAP reflects the gradual release and accumulation of stronger electron-donating compounds. Together, these assays confirm the nanoliposomes’ ability to provide sustained and phase-dependent antioxidant release, offering complementary insights into the release kinetics and mechanisms of action of the encapsulated compounds [2,3,4].

### 2.15. Antioxidant Activity of Nanoliposomal Formulations

The data presented in Table 5 show the antioxidant activity of different treatments, including nanoliposomes loaded with *Dunaliella tertiolecta* extract (Dt-NL), β-carotene-loaded nanoliposomes (β-CAR-NL), unloaded nanoliposomes (NC-NL), free microalgal extract, and commercial β-carotene, evaluated through ABTS•^+^ radical scavenging and ferric reducing antioxidant power (FRAP) assays. The results indicate that all loaded formulations exhibited significantly higher antioxidant activity compared to the unloaded control (NC-NL), which showed no detectable activity in either assay. Among the samples, the microalgal extract and commercial β-carotene in free form demonstrated the highest antioxidant capacities, particularly in the FRAP assay, with values of 47.62 ± 0.88 µmol TE/g and 49.45 ± 0.6 µmol TE/g, respectively. These high FRAP values suggest a potent electron-donating capacity, characteristic of both chlorophyll derivatives and carotenoids present in the microalgal extract, as well as the β-carotene molecule itself.

Interestingly, the Dt-NL formulation exhibited antioxidant activities of 12.44 ± 0.12 µmol TE/g (ABTS•^+^) and 46.11 ± 0.83 µmol TE/g (FRAP), comparable to the free microalgal extract, particularly in the FRAP assay, where no statistically significant differences were observed between Dt-NL and the microalgal extract. In contrast, β-CAR-NL showed slightly lower antioxidant capacity (10.8 ± 0.17 µmol TE/g in ABTS•^+^ and 41.52 ± 1.14 µmol TE/g in FRAP) than Dt-NL, supporting the idea that the presence of a complex mixture of bioactive compounds in the microalgal extract may offer synergistic effects that enhance antioxidant potential beyond what β-carotene alone can achieve. The superior performance of the microalgal extract and Dt-NL in the FRAP assay, which operates exclusively through a single electron transfer (SET) mechanism, suggests a strong capacity for reducing ferric ions, while the ABTS•^+^ assay, sensitive to both SET and hydrogen atom transfer (HAT) mechanisms, revealed moderate but effective radical scavenging activity.

From a bioavailability and functionality standpoint, nanoliposomal encapsulation offers key advantages for protecting sensitive antioxidant compounds like carotenoids and pigments during gastrointestinal transit. The lipid bilayer serves as a barrier against harsh digestive conditions, preserving the structural integrity and antioxidant activity of the encapsulated molecules until their controlled release in the intestine. The amphiphilic nature of nanoliposomes also enhances the solubility and absorption of hydrophobic compounds such as β-carotene, improving bioavailability. Results indicate that Dt-NL maintains comparable antioxidant activity to the free microalgal extract, with the added benefit of sustained protection and gradual release. While the free forms showed slightly higher immediate activity, encapsulation ensures prolonged efficacy during digestion, crucial for functional and nutraceutical applications. This improved performance is attributed to the chemical structure of carotenoids, whose conjugated double bonds and terminal rings enable electron donation and resonance stabilization, preserving their antioxidant activity [54].

### 2.16. Erythroprotective Potential of Loaded Nano-Liposomes

The results presented in Table 6 demonstrate that nanoliposomes loaded with *Dunaliella tertiolecta* extract (Dt-LN) and β-carotene-loaded nanoliposomes (β-CAR-LN) exhibited significantly higher erythroprotective effects compared to unloaded nanoliposomes (UL-N), which showed minimal hemolysis inhibition across all analyzed blood groups (A+, B−, O+, AB+). This finding indicates that the protective effect is directly associated with the presence of bioactive compounds incorporated within the nanoliposomes rather than the liposomal structure itself. Among the evaluated treatments, free β-carotene displayed the highest percentage of hemolysis inhibition, reaching up to 94.70 ± 3.83% in the A+ group and 94.66 ± 3.86% in the O+ group, highlighting its strong antioxidant efficacy in neutralizing free radicals and preserving erythrocyte membrane integrity. However, the microalgal extract also exhibited a considerable antioxidant potential, though lower than that of pure β-carotene, possibly due to the synergistic action of multiple antioxidant compounds present in the algal biomass contributing to hemolysis inhibition.

The mechanism underlying these results involves the inhibition of hemolysis induced by AAPH. This compound generates peroxyl radicals through thermal decomposition, triggering lipid peroxidation in erythrocyte membranes and leading to hemoglobin (Hb) release. Measuring released Hb by spectrophotometry serves as an indirect indicator of oxidative damage, where lower Hb levels indicate stronger anti-hemolytic (erythroprotective) activity. Nanoliposomes enhance the stability and controlled release of encapsulated antioxidants, improving their interaction with AAPH-generated radicals and extending their protective effect. This explains the superior performance of encapsulated versus free compounds, confirming nanoliposomes as effective delivery systems for prolonging antioxidant action and protecting red blood cells from oxidative stress [52,55].

Moreover, it is important to emphasize that the encapsulation of β-carotene and *D. tertiolecta* extract within nanoliposomes allows for sustained release and potentially higher bioavailability at the site of oxidative stress generation, providing continuous protection against radical-induced membrane damage. The differential efficacy between free and encapsulated antioxidants may also be attributed to the release kinetics and the interaction dynamics of the bioactive compounds with the erythrocyte membrane surface, where nanoliposomes facilitate close contact with the cellular targets. This protective strategy reinforces the relevance of designing sustained-release delivery systems to prevent oxidative damage, particularly in sensitive cellular models such as erythrocytes [55].

Regarding the variability observed among different blood groups, it is important to consider that erythrocyte membrane composition differs between A+, B−, O+, and AB+ groups due to the presence of specific surface antigens (agglutinogens), which may influence susceptibility to oxidative damage and the interaction with antioxidant compounds. These structural differences can affect the arrangement of membrane glycoproteins and sphingolipids, modifying the accessibility of free radicals to vulnerable lipid sites. Therefore, evaluating the antioxidant effect and erythroprotective potential across different blood groups provides valuable insight into potential variations in therapeutic response, supporting the development of personalized antioxidant strategies. This approach could be particularly relevant in preventing oxidative stress-related complications in hematological and cardiovascular disorders, considering individual blood group characteristics to optimize nutraceutical formulations and clinical applications [1,3].

### 2.17. Citotoxicity of Loaded Nano-Liposomes

The cytotoxicity results presented in Table 7 reveal notable differences in the percentage of hemolysis induced by the various treatments across the different erythrocyte blood groups. Both nanoliposomes loaded with *Dunaliella tertiolecta* extract (Dt-LN) and β-carotene-loaded nanoliposomes (β-CAR-LN) exhibited moderate cytotoxicity values, generally higher than those observed for unloaded nanoliposomes (UL-N), particularly in blood types A+, B−, and O+. This suggests that while the incorporation of bioactive compounds into nanoliposomal systems enhances their erythroprotective potential, it may also slightly increase membrane interaction, potentially leading to higher hemolytic activity under oxidative conditions. Notably, free β-carotene consistently demonstrated the lowest cytotoxicity values among the treatments in most blood groups, with percentages as low as 2.36 ± 0.85% in A+ and 5.7 ± 0.69% in AB+, highlighting its biocompatibility and safe profile for erythrocyte membranes.

The mechanism underlying these cytotoxicity results is closely linked to oxidative stress-induced hemolysis caused by bioactive compounds. Radical generation attacks the phospholipid bilayer of erythrocyte membranes, promoting lipid peroxidation and structural destabilization, leading to hemoglobin (Hb) release into the extracellular medium. The amount of Hb released, measured spectrophotometrically, indicates the extent of membrane damage, with higher Hb levels reflecting greater oxidative damage and cytotoxicity. While nanoliposome delivery systems enhance the efficacy of antioxidant agents, they may also increase interaction with erythrocyte membranes, potentially elevating oxidative damage depending on the concentration and release rate of encapsulated compounds. Interestingly, the microalgal extract showed cytotoxicity levels comparable to or slightly higher than nanoliposome-encapsulated treatments, especially in B- and O+ blood groups, likely due to other active metabolites in the extract interacting with cell membranes. Variations between free and encapsulated β-carotene highlight how nanoencapsulation modulates bioactivity and membrane interaction, reinforcing the need to optimize nanocarrier systems to balance antioxidant benefits with minimal cytotoxic effects [23,45].

The variability in cytotoxic responses observed among the different erythrocyte blood groups can be explained by the distinct composition and structural organization of membrane surface antigens (agglutinogens) present in each blood type (A+, B−, O+, AB+). These antigens, composed primarily of glycoproteins and glycolipids, influence the biophysical properties of the erythrocyte membrane, including fluidity, charge distribution, and susceptibility to oxidative damage. Such differences may modulate the interaction of bioactive compounds and nanoliposomes with the membrane, leading to varying degrees of hemolytic response. Studying these variations across blood groups is essential for the development of targeted antioxidant therapies and the design of biocompatible delivery systems tailored to individual patient profiles. This approach could have significant clinical applications in personalized medicine, particularly in preventing oxidative damage in transfusion medicine, anemia management, and cardiovascular health, where erythrocyte stability plays a critical role [1,23]**.**

### 2.18. Analysis by Scanning Electron Microscopy (SEM)

**Figure 12:**
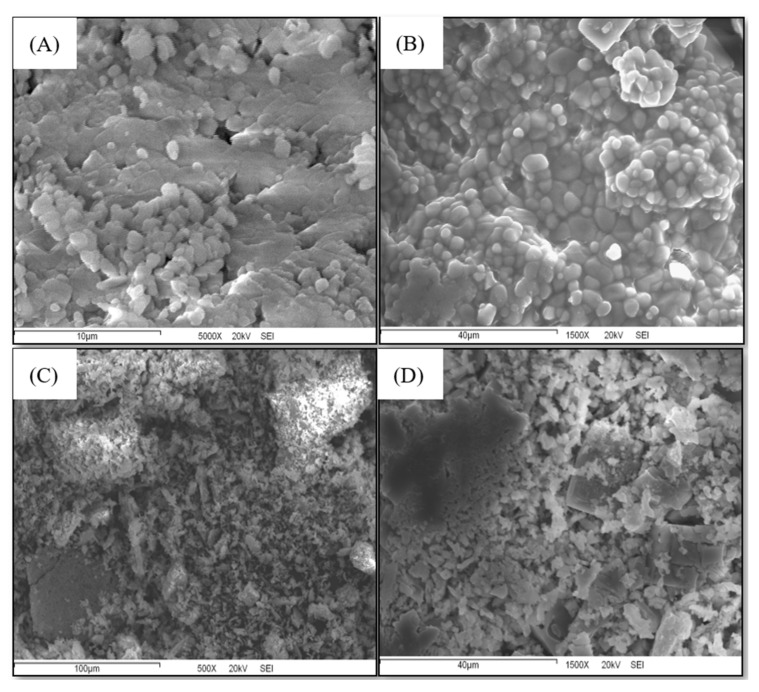
SEM micrographs of nano-liposomes loaded with microalgal extract (**A**,**B**) and β-carotene (**C**,**D**).

Nano-liposomes prepared via the particle dispersion method were observed under a scanning electron microscope to characterize the morphology and shape. Samples were observed in a range of 500× to 5000× magnification to confirm the occurrence of well-formed nano-sized and spherical liposomes. Figure 5 is divided into four images, two corresponding to Dt-NL and two to β-CAR-NL. Figure 12 shows scanning electron microscopy (SEM) micrographs of nanoliposomes loaded with the microalgal extract (Figure 5a,b) and β-carotene (Figure 5c,d), providing detailed insights into the morphology and surface characteristics of the nanoliposomal systems. The images at different magnifications clearly illustrate distinct structural features between the two types of loaded nanoliposomes, which are critical for understanding their release behavior, stability, and functional properties.

In the micrographs of the Dt-LN formulation (Figure 12A,B), nanoliposomes display a uniform spherical to semi-spherical morphology with a dense, compact surface and strong aggregation, suggesting close interaction among lipid vesicles. At higher magnification (5000×), the smoother surface indicates effective encapsulation of the microalgal extract, likely contributing to reduced permeability and a slower diffusion rate of bioactives. These features align with the sustained release observed in antioxidant assays, as the compact structure may act as a barrier to rapid release. In contrast, SEM images of β-CAR-LN (Figure 12C,D) show a more irregular, porous morphology with rough surfaces, visible cracks, and loosely aggregated particles. This less compact structure likely results from interactions between hydrophobic β-carotene and the lipid bilayer, leading to less stable vesicle formation. Consequently, the β-CAR-LN formulation may allow faster release of encapsulated compounds, particularly under digestive conditions where bile salts and enzymes disrupt nanocarrier integrity [16,22].

From a functional perspective, these morphological characteristics have direct implications on the release kinetics, stability, and antioxidant functionality of the nanoliposomal formulations. The denser and smoother surface of Dt-LN suggests enhanced physical stability by limiting oxidative degradation and premature release of the encapsulated compounds, promoting a prolonged and controlled release profile as confirmed by the ABTS and FRAP assays. This structural integrity is essential for protecting sensitive bioactives like chlorophylls and carotenoids throughout processing, storage, and gastrointestinal transit. On the other hand, the β-CAR-LN system, with its more porous and less cohesive morphology, may exhibit faster release kinetics due to the easier penetration of aqueous media into the vesicle core and potential structural collapse under physiological conditions. While this may lead to higher initial bioavailability, it could also compromise long-term stability and result in faster degradation of β-carotene, which is highly susceptible to oxidation when exposed to environmental factors [22].

SEM analysis confirms that morphological differences between Dt-LN and β-CAR-LN nanoliposomes significantly influence their release behavior, stability, and antioxidant performance. The compact, uniform structure of Dt-LN supports prolonged delivery and sustained antioxidant activity, making it more suitable for functional food and nutraceutical applications. In contrast, the β-CAR-LN formulation, with its porous, irregular morphology, may require optimization to improve structural integrity and control release. Size analysis revealed that Dt-LN nanoliposomes mainly ranged between 200–500 nm with consistent, compact morphology, while β-CAR-LN showed greater heterogeneity, with some vesicles reaching 600 nm and forming irregular aggregates. This smaller, more homogeneous size distribution in Dt-LN aligns with enhanced stability and a sustained-release profile. Conversely, the larger, less cohesive β-CAR-LN structures may facilitate faster diffusion but could compromise long-term stability and protection of the encapsulated bioactives. These findings underscore the importance of morphological characterization in designing effective nanocarrier systems [16,22].

### 2.19. Characterization Using Fourier Transform Infrared Spectroscopy (FT-IR)

**Figure 13:**
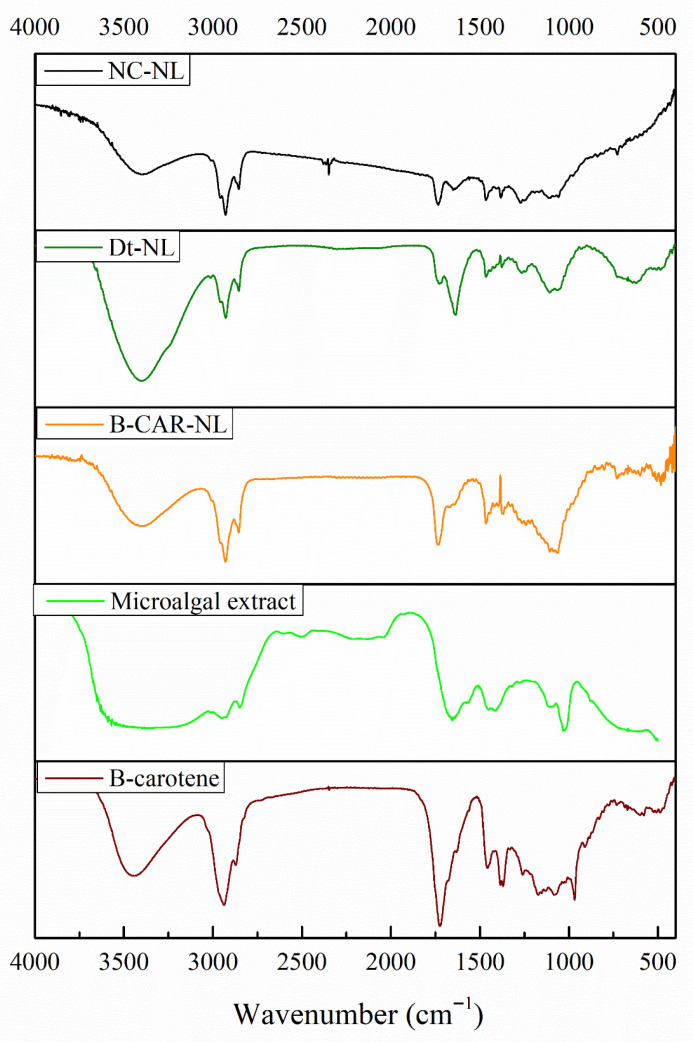
Fourier transform infrared (FT-IR) spectra of NC-NL (unloaded nanoliposomes), Dt-NL (nanoliposomes loaded with *Dunaliella tertiolecta* extract), β-CAR-NL (β-carotene-loaded nanoliposomes), microalgal extract, and commercial β-carotene.

The Fourier-transform infrared (FT-IR) spectra presented in Figure 13 provide a comparative analysis of the functional group composition across unloaded nanoliposomes (NC-LN), nanoliposomes loaded with *Dunaliella tertiolecta* extract (Dt-LN), β-carotene-loaded nanoliposomes (β-CAR-LN), the microalgal extract, and commercial β-carotene. In the β-carotene spectrum (11E), characteristic vibrational bands are clearly observed around 3020–3000 cm^−1^ (C–H stretching of aromatic rings) and near 1600 cm^−1^ (C=C stretching of conjugated polyene chains), confirming the presence of its highly conjugated hydrocarbon backbone. Similar bands appear in the microalgal extract (11D), supporting the presence of carotenoids such as β-carotene and xanthophylls (e.g., lutein, violaxanthin). Additional broad O–H stretching bands around 3650–3300 cm^−1^ in the microalgal extract suggest the coexistence of hydroxylated carotenoids (xanthophylls), possibly along with other polar compounds like chlorophyll derivatives or phenolics, not present in the pure β-carotene spectrum.

In the Dt-NL spectrum, the presence of these characteristic carotenoid bands is preserved, confirming successful encapsulation of pigments into the lipid matrix. The O–H stretching band is broadened, and additional signals in the region between 1700 and 1735 cm^−1^ (C=O stretching) appear, likely due to ester linkages from the phospholipid bilayers of the nanoliposomes interacting with the encapsulated compounds. This feature is shared with the NC-NL and β-CAR-NL spectra, indicating the structural contribution of the phospholipid components. However, Dt-NL and β-CAR-NL display a more complex spectral pattern compared to NC-NL, confirming the successful incorporation of bioactive molecules within the vesicles. Notably, β-CAR-NL exhibits weaker O–H signals, consistent with the non-polar nature of β-carotene, and shows C=O stretching signals due to the liposomal structure rather than from the carotenoid itself [18,27].

Structurally and molecularly, the preservation of the carotenoid-related signals, particularly the conjugated C=C stretching (~1600 cm^−1^) and aromatic C–H (~3020–3000 cm^−1^) bands, alongside lipid-associated ester (C=O) peaks, supports the integrity of the encapsulated β-carotene and microalgal carotenoids within the nanoliposomal systems. The broader O–H signals in Dt-NL and the microalgal extract reflect the complex nature of these matrices, which may include xanthophylls and other polar metabolites that could influence the antioxidant capacity and release behavior of the formulations. The FT-IR data validate the encapsulation process, confirm the presence of key functional groups associated with carotenoids, and suggest potential molecular interactions between the encapsulated pigments and the lipid bilayer, which may contribute to the controlled release and stability observed in the antioxidant assays [27,55].

FT-IR spectroscopy is a key tool for characterizing nanoliposomal systems, offering insights into functional groups and molecular interactions between encapsulated bioactives and the lipid matrix. It confirms successful encapsulation by identifying characteristic vibrational bands of both the active compounds and phospholipid bilayers, providing evidence of chemical stability and potential bonding. In this study, FT-IR verified the incorporation of carotenoids, including β-carotene and pigments from *Dunaliella tertiolecta*, by detecting bands associated with conjugated double bonds (C=C), aromatic C–H stretching, and ester carbonyl groups (C=O). Spectral differences between unloaded and loaded nanoliposomes suggested interactions with the lipid bilayer, relevant to encapsulation efficiency, stability, and release behavior. This structural validation supports the integrity and functionality of the nanoliposomes, enhancing understanding of their performance in antioxidant delivery applications [18].

The FT-IR analysis presented in Table 8 provides a detailed identification of the functional groups present in the loaded nanoliposomes (Dt-NL, β-CAR-NL, NC-NL), the microalgal extract of *Dunaliella tertiolecta*, and commercial β-carotene. The presence of characteristic vibrational bands in the range of ~3020–3000 cm^−1^ (C–H stretching of aromatic rings) and ~1600 cm^−1^ (C=C stretching) confirms the existence of conjugated double bond systems, which are typical of carotenoids. In particular, the identification of these bands in both the Dt-NL formulation and the microalgal extract suggests the presence of carotenoid pigments with polyene chains, including β-carotene, lutein, and violaxanthin, all of which are commonly synthesized by *D. tertiolecta*. The detection of O–H stretching bands (~3650–3300 cm^−1^) across all samples corresponds to alcohol functional groups, likely related to hydroxylated carotenoids (xanthophylls) such as lutein or zeaxanthin, and to possible residual water content or hydroxyl groups from the phospholipid bilayers of the nanoliposomes. Additionally, the presence of C=O stretching signals between 1730–1700 cm^−1^, particularly in NC-NL and Dt-NL, can be associated with ester groups from phospholipid molecules or esterified carotenoids [18,51].

Structurally, the identification of N–H stretching (3500–3180 cm^−1^), C–N (~1200 cm^−1^), and amide N–H bending (~1640 cm^−1^) bands in Dt-NL and the microalgal extract suggests interactions between amino acid residues or protein-like components and the encapsulated pigments, supporting the notion of a complex extract composition. The detection of alkene C=C conjugated systems (~1640 cm^−1^) further confirms the predominance of polyunsaturated carotenoids with extensive π-electron delocalization, which is a hallmark of β-carotene and related compounds. Importantly, the alkane C–H (~2950–2800 cm^−1^) and C–H_2_ (~1450 cm^−1^) stretching bands observed across all samples reflect the lipidic nature of the nanocarriers. The presence of esters (C=O 1750–1735 cm^−1^) specifically in NC-NL, β-CAR-NL, and commercial β-carotene also aligns with the molecular structure of β-carotene derivatives and the phospholipid matrix. Moreover, the identification of alkyne C–H stretching (~3300 cm^−1^) and nitro groups (-NO_2_ at ~1390 cm^−1^) in the microalgal extract and Dt-NL may indicate additional complex metabolites, including oxidized or nitrogen-containing carotenoid derivatives. Overall, the FT-IR results confirm the presence of β-carotene in the D. tertiolecta extract, together with other xanthophylls, as evidenced by the characteristic polyene and aromatic signals. These findings not only support the structural diversity of the pigment profile but also validate the successful encapsulation of these bioactives within the nanoliposomal systems [18,27].

Building on this structural confirmation, the nanoliposomal systems developed in this study demonstrated enhanced stability, encapsulation efficiency, and controlled release behavior, which collectively highlight their potential for translational applications. The antioxidant and erythroprotective effects observed reinforce their suitability for use in functional foods and nutraceuticals, where oxidative stability and bioavailability are critical determinants of efficacy. Moreover, their capacity to provide sustained protection against oxidative stress underscores opportunities for biomedical applications, particularly in preventing or mitigating cellular damage associated with redox imbalance. Taken together, the integration of *Dunaliella tertiolecta* extract and pure β-carotene into nanoliposomal carriers offers a versatile delivery strategy with broad applicability across nutrition, health, and therapeutic contexts.

## 3. Materials and Methods

### 3.1. Chemical Reagents

The chemical reagents employed throughout this research included food-grade β-carotene, soy phosphatidylcholine (SPC), cholesterol, DPPH (1,1-diphenyl-2-picrylhydrazyl), ABTS [2,2′-azinobis (3-ethylbenzothiazoline)-6-sulfonic acid], dimethyl sulfoxide (DMSO), sodium acetate buffer, ferric chloride (FeCl_3_), TPTZ (2,4,6-tripyridyl-s-triazine), Triton X-100, AAPH [2,2′-azobis (2-methylpropionamidine) dihydrochloride], and phosphate-buffered saline (PBS). All reagents were procured from Sigma-Aldrich Co., Ltd. (St. Louis, MO, USA). Additionally, the solvents used for the different analytical procedures were of analytical grade and ensured to be of the highest commercially available purity.

### 3.2. Biological Material and Ethical Considerations

All experimental procedures involving human red blood cells (RBCs) were performed following international ethical standards and regulations, including the FDA guidelines [58] (CFR—Code of Federal Regulations Title 21, Part 640, Subpart B: Red Blood Cells, Section 640.14 Blood Tests [21 CFR 640.14]), as well as the Official Mexican Standard NOM-253-SSA1-2012 [59], which specifies the requirements for the collection, processing, and therapeutic use of human blood and its components. The erythrocyte membranes utilized in this study were obtained from the clinical analysis laboratory at the University of Guadalajara, a facility accredited under ISO-IEC 17025 (NMX-EC-17025) [60] and ISO 15189 standards [61], developed by the ISO/TC 212 [62] technical committee on clinical laboratory testing and in vitro diagnostic systems. These accreditations, along with ISO/IEC 17025 and ISO 9001 [63], ensure the quality and reliability of the procedures followed [60,61,62].

Blood samples were collected from healthy adult volunteers aged between 20 and 40 years, with an erythrocyte concentration ranging from approximately 4.7 to 6.1 × 10^6^ cells/μL. Before blood collection, all participants provided written informed consent. Venous blood sampling was performed using sterile techniques, with EDTA employed as an anticoagulant to preserve the red blood cells [63,64]. The research protocol was reviewed and approved by the corresponding institutional ethics committee (Approval Code: CI 2023-47).

### 3.3. Microalgae Strain and Growth Kinetics of Dunaliella tertiolecta

The marine microalgae *Dunaliella tertiolecta* was obtained from the strain collection of the “Department of Scientific and Technological Research, University of Sonora (DICTUS)”. The bioassays were conducted in 1 L Erlenmeyer flasks containing 700 mL of culture medium, under controlled conditions of temperature (20 ± 1 °C) and light intensity (274 ± 52.5 μmol photons m^−2^ s^−1^). The cultures were maintained using the F/2 medium, as described by Ryther and Guillard [65], which contains nitrates, phosphates, silicates, trace metals, and vitamins.

Table 9 summarizes the different culture media employed to evaluate the microalgae’s growth behavior and adaptation under various cellular stress conditions, including nitrogen deprivation and varying salinity levels. The standard F/2 medium, prepared with 0.88 mol·L^−1^ sodium nitrate (NaNO_3_) at 35 practical salinity units (PSU), is widely recognized as the optimal medium for microalgae cultivation in aquaculture research. It is important to note that these experimental treatments were exclusively applied for the assessment of growth kinetics.

The growth kinetics of *Dunaliella tertiolecta* were evaluated under culture conditions using the optimized medium specifically designed to enhance both microalgal growth and the production of antioxidant pigments. The microalgae cultures were monitored over a period of 21 days, covering all growth phases. To determine the growth kinetics, 1 mL of culture from each treatment was collected daily. Lugol’s solution (I_2_ 1% and KI 2% in distilled water) was added to each sample to fix the cells and facilitate counting. The fixed samples were then loaded into a Neubauer hemocytometer (0.1 mm depth). Cell enumeration was performed using a compound optical microscope (Olympus CX43, Olympus Europe, Hamburg, Germany). Cell density was calculated according to Equation (7), following the methodology described by Goiris et al. [5].

(7) $$\left(\#\frac{Cells}{8}\right)\times 10,000=\frac{\#Cells}{\mathrm{mL}}$$

### 3.4. Experimental Design and Statistical Optimization for Pigment Production

To optimize pigment production, a central composite design (CCD) was employed, with a minimum of three replicates per treatment (*n* ≥ 3). The interactions between the independent variables at different levels were systematically evaluated. The independent variables included low-nitrogen concentration media (0.22 and 0.88 mol·L^−1^), varying salinity levels (25, 35, and 45 PSU), and culture age (harvest day). The CCD was structured based on a second-order polynomial model (Equation (8)):

(8) $$Y=\beta_{0}\sum_{i=1}^{k}\beta_{i}X_{i}+\sum_{i=1}^{k}\beta_{ii}X_{ii}^{2}+\sum_{i}^{k-1}\sum_{j}^{k}\beta_{ij}X_{i}X_{j}+ɛ$$

where Y represents the response variable, β_0_ is the intercept, βₖᵢ denotes the linear regression coefficients, βₖᵢᵢ represents the quadratic regression coefficients, βₖⱼᵢ corresponds to the interaction coefficients between variables, and ɛ is the experimental error term. Culture conditions were optimized to maximize pigment production and enable accurate quantification. The responses analyzed using Equation (8) included chlorophyll a, chlorophyll b, chlorophyll c, total chlorophyll, total carotenoids, and overall pigment yield.

The optimization matrix was constructed with an integrated ANOVA (analysis of variance) using JMP V18, considering statistical significance at *p* < 0.05. Following matrix generation, a lack-of-fit test (*p* > 0.05) was performed to confirm the adequacy of the model and to ensure that the predicted values appropriately fit the experimental data. The relationship between the independent variables and the responses was modeled using Equation (2). The total number of experimental runs (N) required for the CCD was determined using the equation:

N = 2^k (k − 1) + C_0_

where k represents the number of factors and C_0_ denotes the number of replicates at the central point of the design. CCD was specifically chosen due to its efficiency in reducing the number of experimental runs required when three variables are involved. Based on the variance analysis, regression coefficients for linear, quadratic, and interaction terms were calculated [24].

### 3.5. Response Surface Methodology (RSM)

Response Surface Methodology (RSM) was applied to evaluate the influence of nitrogen concentration (NC), salinity, and culture age (AC) on six response variables: chlorophyll a, chlorophyll b, chlorophyll c, total chlorophyll, total carotenoids, and overall pigment yield. Overlay plots were used as part of the optimization technique to generate response surface graphics. To visualize the relationships between the experimental factors and the responses, regression coefficients derived from the fitted polynomial equation were used to produce three-dimensional surface plots. These plots facilitated the identification of optimal conditions for pigment production. All statistical analyses, including the generation of 3D surface plots, were performed using the JMP V11 software package.

### 3.6. Pigment Extraction and Quantification

For pigment extraction, 200 mL of microalgae culture was harvested and centrifuged at 3200× *g* for 10 min using a Thermo Scientific™ Heraeus™ Multifuge™ X1R centrifuge (Thermo Fisher Scientific, Waltham, MA, USA). The supernatant was discarded, and the resulting pellet was immediately frozen at –80 °C, followed by lyophilization using a Yamato Scientific Co., Ltd. (Chuo City, Japan) DC401 freeze dryer.

Once the biomass was completely dried, 5 mL of methanol (99%) was added, and the mixture was stored at 4 °C in the dark for 24 h. Pigment extraction was performed using ultrasound-assisted extraction with a Branson Digital Sonifier (Osonica LLC, Newtown, CT, USA), applying three 15-s pulses at 30% amplitude (400 W, 500 MHz) to disrupt the cell walls and release the intracellular pigments. After sonication, the samples were again stored at 4 °C in the dark for an additional 24 h. Subsequently, the samples were centrifuged at 3200× *g* for 10 min. Pigment concentrations were determined by measuring absorbance at specific wavelengths corresponding to each pigment’s absorption spectrum. Chlorophyll a, chlorophyll b, chlorophyll c, total chlorophyll, and total carotenoids were quantified following the methods described by Lichtenthaler and Wellburn [27].

Before pigment quantification, the extraction protocol was standardized using 99% methanol as the solvent. The concentrations of chlorophyll a (Ca), chlorophyll b (Cb), chlorophyll c (Cc_1_ + Cc_2_), total chlorophyll (Ctotal), and total carotenoids (Cx + c) were calculated based on their respective absorbance values. Measurements were conducted using 300 μL of the methanolic extract loaded into a 96-well microplate, read with a Multiskan Go microplate spectrophotometer (Thermo Scientific, Waltham, MA, USA). Pigment concentrations were initially obtained in μg/mL and subsequently expressed as mg/g dry weight (dw), according to the following Equation (9):

C*_a_* = 11.85A_664_ − 1.54A_647_ + 0.08A_630_ C*_b_* = −5.43A_664_ + 21.03A_647_ − 2.66A_630_ C*_c1+c2_* = −1.67A_664_ − 7.6A_647_ + 21.52A_630_ C*_total_* = 21.3877A_630_ + 10.3739A_647_ + 10.3739A_664_ + 5.5309A_691_ C*_x+c_* = (1000.65A_470_ − 2.86C_a_ − 129.2C_b_)/221 (9)

Following the optimization process, pigments were extracted from the cultures grown under the selected optimal conditions, the microalga was harvested through centrifugation (Thermo Scientific^TM^, Heraeus^TM^ modelo Multifuge^TM^ X1R) and decantation until a pellet was obtained. It was then frozen and subjected to a lyophilization process (Yamato Scientific Co., Ltd lyophilizer, model DC401). After obtaining the lyophilized microalga, 90% ethanol was added and left to rest for 24 h in refrigeration and darkness. Finally, ultrasonic pulses were applied to disrupt the cell membrane and completely release the pigments into the solvent. The ethanolic extract was stored in darkness and refrigerated at 4 °C until its encapsulation in nano-liposomes. The concentration of the extract was determined by weight comparison between an empty centrifuge tube (initial weight) and crude extract after ethanol evaporation (final weight) via direct nitrogen gas application.

### 3.7. High-Performance Liquid Chromatography (HPLC)

The determination of carotenoids was performed using a high-performance liquid chromatography (Agilent Technologies 1260 Infinity, Santa Clara, CA, USA), connected in series to a diode array detector (DAD). Before the analysis, the microalga extract was dissolved in acetone. The carotenoids were separated on a reverse-phase C_18_ column using a linear gradient of acetone as the mobile phase. The spectra were measured between 200 and 600 nm, and the chromatograms were processed at 475 nm [12,42].

### 3.8. Preparation of Nano-Sized Liposomes via the Particle Dispersion Method

Nano-liposomes were formulated with L-α-phosphatidylcholine (SIGMA-ALDRICH, St. Louis, MO, USA, P7443 from soybean), cholesterol (SIGMA-ALDRICH, C8667-5G), and microalgae extract, as well as commercial β-carotene in a 1:1:1 ratio, using 99% chloroform as suspension medium and solvent. β-carotene was obtained commercially (SIGMA-ALDRICH, C9750-10G), and the green pigmented extract of *D. tertiolecta* was used for encapsulation separately.

A total of 60 mg of phosphatidylcholine and cholesterol was weighed and dissolved in 25 mL of 90% chloroform. The mixture was then agitated with vortex equipment for 30 s, followed by centrifugation for 10 min at 1500 rpm. Once the mixture was obtained, it was incubated at 40 °C for 15 min with constant agitation and then subjected to sonication in a water bath heating for 30 min. Next, 3 mL of the microalgal extract at a concentration of 20 mg/mL was added, homogenized with a vortex, and the incubation and sonication processes were repeated.

A round-bottom flask was weighed to determine the initial weight, and the mixture was poured into it. A rotary evaporator was used to remove the solvent and obtain a pellet, which was then resuspended in 50 mL of saline solution to form micelles. The mixture was vortexed, incubated, and sonicated twice after the re-suspension. Once the liposomes were formed, ultrasonic pulses were applied in three intervals of 5 min at 30% power, 400 W, and 500 mHz (Generator ultrasonic pulses Branson Digital Sonifier Osonica, LLC., E.U.A., Danbury, CT, USA) to reduce their size to the nanoscale. Finally, the suspension of nano-liposomes in saline solution was frozen and stored in darkness for further lyophilization to obtain a dried nano-liposome product [27].

### 3.9. Encapsulation Efficiency Analysis

Encapsulation efficiency was determined using an extraction technique based on the protocol described by Pan et al. [18], with slight modifications. Briefly, 400 µL of β-carotene-loaded nanoliposome solution and D. tertiolecta extract-loaded nanoliposome were mixed with 1 mL of petroleum ether and stirred continuously at 45 rpm for 5 min at 30 °C. The mixture was then centrifuged at 4000× *g* for 5 min to recover the supernatant. The upper phase was collected and subjected to rotary evaporation to remove the petroleum ether. The resulting dry residue was redissolved in chloroform, and the amounts of free *D. tertiolecta* extract and β-carotene were quantified by UV–Vis spectrophotometry at 450–452 nm (carotenoids, especially β-carotene) and ~665 nm (chlorophyll a), using a 96-well microplate reader (Multiskan GO, Thermo Fisher Scientific Inc., New York, NY, USA). Encapsulation efficiency (%) was calculated using the following equation:

(10) $$Encapsulation\;Efficiency=\;\frac{{Sample}_{total}-{Sample}_{free}}{{Sample}_{total}}\times \;100$$

### 3.10. Assessment of Particle Size and Zeta Potential Stability

Aliquots of liposomal dispersions (1.0 mL) were diluted in 100 mL of phosphate buffer and subsequently transferred into polystyrene cuvettes. The hydrodynamic diameter (Dz) and zeta potential (ζ) were determined with a Nano-ZS90 particle size analyzer (Malvern Instruments Ltd., Malvern, UK). Each measurement was performed in triplicate to ensure reproducibility.

### 3.11. Assessment of Centrifugal Stability

The stability of the nanoliposome samples was assessed following the method described by Ghorbanzade et al. [51], with minor modifications. Briefly, 5 mL of the nanoliposome suspension was centrifuged at 3500× *g* for 15 min. Nanoliposome stability (NS) was determined using the following equation:

(11) $$NS=\frac{F_{ev}}{I_{ev}}\times 100$$

### 3.12. Scanning Electron Microscopy (SEM)

Scanning electron microscopy was used to observe the topography and surface morphology of the loaded nano-liposomes. Nano-liposomes were freeze-dried prior to analysis for liposome particle sizes and shapes [66].

### 3.13. Method for In Vitro Release Study

The in vitro release studies were conducted to evaluate the sustained-release behavior of both β-carotene and D. tertiolecta extract when encapsulated in nanoliposomes. For this purpose, 10 mL of β-carotene solution, β-CAR-LN, D. tertiolecta extract, and Dt-LN—each containing 1 mg of the respective compound—were placed separately into dialysis membranes (molecular weight cutoff: 8000–14,000 Da). The membranes were immersed in 100 mL of phosphate-buffered saline (PBS, 0.05 mol/L, pH 7.4) and maintained at 37 °C under constant agitation at 100 rpm to simulate physiological conditions. At predetermined time intervals (0.5, 1, 2, 4, 6, 8, 10, 12, and 24 h), 1 mL aliquots of the release medium were withdrawn and immediately replaced with fresh PBS to maintain sink conditions. The concentration of β-carotene was determined at 460 nm, while that of the D. tertiolecta extract was quantified at 462 nm, based on prior spectral scanning that identified absorption maxima characteristic of carotenoids (453, 462, and 478 nm). Individual calibration curves for β-carotene and the extract were prepared to ensure accurate quantification. The release data were expressed as the percentage of compound released over time. All experiments were performed in triplicate. The release percentage was calculated using the following equation:

(12) $$Releaserate=\frac{Released}{TotalFXN}\times 100$$

The release kinetics of the encapsulated compound were evaluated using the Korsmeyer–Peppas model. The concentration of the released compound (M_t_) at various time points (t) was determined by UV–Vis spectrophotometry. The normalized release fraction (M_t_/M_∞_) was then calculated, where M_∞_ represents the total amount released at the end of the experiment (24 h). The data were fitted to the model equation:

(13) $$\frac{M_{t}}{M_{\infty }}=k\times t^{n}$$

where *k* is the kinetic constant and *n* is the release exponent. Parameter fitting was performed using nonlinear regression with statistical software (JMP v18), and the goodness of fit was assessed by the coefficient of determination (R^2^). This analysis allowed for the identification of the predominant release mechanism (Fickian diffusion, anomalous transport, or matrix relaxation) for each evaluated system.

### 3.14. Sustained-Release Kinetics and Antioxidant Activities

The sustained-release kinetics of nanoliposomes loaded with microalgal biomass extract and β-carotenoid were evaluated under simulated physiological conditions by incubating the nanoliposomal suspension at 37 °C with constant stirring. Samples were collected at predefined time intervals, and the amount of released bioactive compounds was quantified using UV–Vis spectrophotometry. Antioxidant capacity of both the encapsulated and released fractions was determined using the ABTS radical scavenging assay and the ferric reducing antioxidant power (FRAP) method.

#### 3.14.1. ABTS^•+^ Assay

The methodology proposed by Re et al. [56], with some modifications, was followed. The 2,2′-azinobis-(3-ethylbenzothiazoline)-6-sulfonic acid cationic radical (ABTS•^+^) was activated by adding potassium persulfate, which oxidized the ABTS agent. The ABTS•^+^ solution was diluted with ethanol to achieve an absorbance of 0.7 ± 0.01 at 734 nm. A control sample was prepared with 20 µL of ethanol + 270 µL of ABTS^•+^, while the test samples (*n* ≥ 3) consisted of 20 µL of nano-liposomes loaded with microalgae extract (Dt-NL) and β-carotene (β-CAR-NL) + 270 µL of ABTS•^+^, individually. The samples were left to rest for 30 min in darkness before being read in triplicate at 734 nm using a 96-well microplate spectrophotometer (Multiskan Go, Thermo Scientific, Waltham, MA, USA).

#### 3.14.2. Ferric Reducing Antioxidant Power Assay

The reducing power of the loaded nano-liposomes was determined using the FRAP assay (Ferric Reduction Antioxidant Power) described by Brand-Williams et al. [57] with some modifications. The stock solutions were sodium acetate buffer (300 mM, pH 3.6), ferric chloride (FeCl_3_) (20 mM), and TPTZ solution (2,4,6-tripyridyl-s-triazine) (10 mM) in HCl (40 mM). The working FRAP solution was prepared in a 10:1:1 ratio (buffer: FeCl_3_). A control sample was prepared with 20 µL of ethanol + 280 µL of FRAP, while the test samples (*n* ≥ 3) consisted of 20 µL of Dt-NL and β-CAR-NL + 280 µL of FRAP, individually. The reaction was left for 30 min in the dark, then read in triplicate at 638 nm using a 96-well microplate spectrophotometer (Multiskan Go, Thermo Scientific, Waltham, MA, USA).

### 3.15. Ethical Handling of Human Erythrocyte Membrane-Based Assays

All procedures involving human red blood cells (RBCs) were conducted in accordance with current international regulations, including those established by the U.S. Food and Drug Administration (FDA) in the Code of Federal Regulations (Title 21, Part 640, Subpart B—Red Blood Cells, Section 640.14), as well as the Official Mexican Standard NOM-253-SSA1-2012, which governs the collection, processing, and therapeutic use of blood and its components. The erythrocyte membranes used as the experimental model were provided by the clinical analysis laboratory at the University of Guadalajara, which holds current accreditations under ISO/IEC 17025 (NMX-EC-17025) and ISO 15189, issued by the ISO/TC 212 committee, specialized in clinical laboratory testing and in vitro diagnostic systems, based on ISO/IEC 17025 and ISO 9001 standards. Blood samples were collected from healthy adult volunteers aged between 20 and 40 years, with erythrocyte counts ranging from 4.7 to 6.1 × 10^6^ cells/µL. All participants signed informed consent forms prior to sample collection. Venous blood was drawn using sterile tubes containing EDTA as an anticoagulant. The isolated erythrocyte membranes were used to evaluate the erythroprotective activity of the sample. The study was carried out under the institutionally approved protocol (CI 2023-47). “All experiments were performed in triplicate (*n* > 3) using erythrocytes from at least 20 healthy adult donors per blood group”.

### 3.16. Antihemolytic Activity of Loaded Nano-Liposomes

To determine the protective activity against oxidative damage in erythrocytes, the 2,2′-azobis-(2-methylpropionamidine) (AAPH) method described by Ruiz-Cruz et al. [64] was used. Five milliliters of blood types A+, O+, B−, and AB+ were extracted through the punction technique from apparently healthy individuals over 18 years old. One milliliter of blood was mixed with 3 mL of saline solution by inversion. Once mixed, the samples were centrifuged at 1500 rpm for 10 min at 25 °C. The plasma was removed, and the inversion and centrifugation process was repeated two more times. Five milliliters of saline solution were taken to combine with 100 µL of washed, non-coagulated blood.

Control solutions (+) (300 µL of erythrocytes), control (−) (150 µL of erythrocytes + 150 µL of AAPH), and test samples (100 µL of erythrocytes + 100 µL of AAPH + 100 µL of Dt-NL and β-CAR-NL) were prepared in 1.7 mL microtubes. The samples were incubated under agitation for 3 h at 37 °C. After the incubation, the samples were suspended in 1 mL of saline solution and centrifuged at 1500 rpm for 10 min at 25 °C. Three hundred microliters of each sample were placed in triplicate at 540 nm in a 96-well microplate for readings (Multiskan Go, Thermo Scientific, Waltham, MA, USA).

### 3.17. Citotoxicity

To determine the cytotoxicity activity in erythrocytes, red blood cell (RBC) samples were collected by venipuncture into EDTA-containing tubes using the Vacutainer system. Blood was obtained from groups A+, O+, B−, and AB+ from apparently healthy individuals over 18 years. A 2% erythrocyte suspension was prepared from each blood group by washing the cells with physiological saline solution (PSS) until complete removal of plasma. Subsequently, 3000 µL of saline was added to 1000 µL of blood, and the mixture was centrifuged at 1500 rpm for 10 min. After centrifugation, the supernatant was carefully removed, and the washing process was repeated three times to ensure complete plasma elimination [67,68].

Control solutions (+) (200 µL of erythrocytes suspension + 100 µL of physiological solution), control (−) (100 µL of erythrocytes suspension + 100 µL of physiological solution + 100 µL of triton 1%), and test samples (100 µL of erythrocytes suspension + 100 µL of physiological solution + 100 µL of Dt-NL and β-CAR-NL) were prepared in 1.7 mL microtubes. The samples were incubated under agitation for 3 h at 37 °C. After the incubation, the samples were suspended in 1 mL of saline solution and centrifuged at 1500 rpm for 10 min at 25 °C. Three hundred microliters of each sample were placed in triplicate at 540 nm in a 96-well microplate for readings (Multiskan Go, Thermo Scientific, Waltham, MA, USA).

### 3.18. Fourier Transform Infrared Spectroscopy (FT-IR)

Interactions between microalgae extract and β-carotene with phosphatidylcholine and cholesterol, forming the nano-liposomes micelles, were evaluated via Fourier transform infrared spectroscopy. FT-IR spectra were recorded with KBr mixed with the samples in pellets form between 4000 and 500 cm^−^^1^ [18].

### 3.19. Statistical Analysis

Statistical analysis of the data was conducted using JMP V18. Data were reported as mean ± standard deviation. Analysis of variance (ANOVA) was conducted, and significant differences between treatments were obtained via Tukey’s multiple comparison tests at a significance level of *p* < 0.05.

## 4. Conclusions

The present study demonstrates that nanoliposomal encapsulation of *Dunaliella tertiolecta* extract and β-carotene represents an effective and innovative strategy for enhancing the functional delivery of natural antioxidants with erythroprotective capacity. The development of these liposomal carriers offers a promising approach to overcome key limitations associated with the low stability, poor solubility, and reduced bioavailability of carotenoids and chlorophylls during processing and physiological conditions. By integrating optimized microalgal cultivation for bioactive enrichment with nanotechnology-based delivery systems, this research contributes to the advancement of encapsulation methodologies capable of sustaining the release of bioactives while preserving their biological activity over time.

The release kinetics revealed an anomalous transport mechanism for both systems, with β-carotene showing a faster and more efficient release due to its higher lipophilic compatibility with the nanoliposomal matrix. Importantly, the study provides novel insights into the synergistic behavior of complex pigment mixtures and their role in mitigating oxidative stress at the cellular level. The erythroprotective effect observed in the nanoencapsulated systems highlights their potential as protective agents against redox imbalance and membrane damage, proposing these nanocarriers as viable candidates for the development of next-generation nutraceuticals, functional foods, or therapeutic adjuncts. This work also acknowledges certain limitations, such as the absence of in vivo validation and the restricted comparison with conventional encapsulation systems, which should be addressed in future research. Nonetheless, the methodological design of this study, combining pigment production optimization and advanced delivery systems, reinforces the relevance of microalgae as sustainable and high-value bioactive sources.

Nanoliposomes containing *Dunaliella tertiolecta* extract and pure β-carotene were successfully developed, showing good encapsulation efficiency, colloidal stability, and controlled release. These properties enhanced their antioxidant and erythroprotective activity, confirming their value as delivery systems for carotenoid-rich bioactives. The findings highlight their potential for application in functional foods, nutraceuticals, and biomedical products, where sustained release and improved bioavailability can provide continuous protection against oxidative stress. Future perspectives involve in vivo evaluation, incorporation into real food matrices or biomedical formulations, and assessment of safety, stability, and scalability. These steps are essential for translating the current results into targeted antioxidant therapies and preventive strategies against oxidative stress-related disorders, thereby broadening the applicability of this nanoencapsulation platform in nutrition and health sciences.

## Figures and Tables

**Table 1:** Central Composite Design for the Optimization of Chlorophyll a, b, and c Production in *Dunaliella tertiolecta*.

	Experimental Variables				Responses (mg/g dw)			Predicted Responses (mg/g dw)		
Run	Pattern	*X*_1_	*X*_2_	*X*_3_	C_a_	C_b_	C_c1+c2_	C_a_	C_b_	C_c1+c2_
1	0A0	0.55	45	10	1.73	0.78	0.46	0.71	0.66	0.09
2	00a	0.55	35	5	1	0.73	0.12	1.17	0.95	0.23
3	a00	0.22	35	10	1.85	1.42	0.52	2.48	1.17	0.93
4	−++	0.22	45	15	0.66	0.39	0.18	1.61	0.48	0.3
5	0a0	0.55	25	10	1.95	1.31	0.45	3.83	1.69	0.98
6	00A	0.55	35	15	0.72	0.48	0.23	0.71	0.53	0.27
7	000	0.55	35	10	1.25	0.73	0.31	0.4	0.45	0.15
8	++−	0.88	45	5	6.52	4.6	1.93	2.32	1.29	0.65
9	+−+	0.88	25	15	2.38	1.77	0.77	2.71	1.54	0.83
10	--+	0.22	25	15	13.68	4.85	3.71	12.29	4.92	3.41
11	+−−	0.88	25	5	4.41	2.24	1.02	3.25	2.08	0.86
12	+++	0.88	45	15	2.88	1.28	0.56	2.32	1.29	0.65
13	−−−	0.22	25	5	6.11	2.66	1.9	6.46	2.58	1.78
14	A00	0.88	35	10	10	1.01	0.63	1.23	1.32	0.65
15	−+−	0.22	45	5	1.15	0.63	0.28	0.61	0.79	0.18

Experimental variables as the independent variables: X_1_; nitrogen concentration (mol·L^−1^ NaNO_3_). X_2_; Salinity (PSU). X_3_; Culture age (days). Responses (mg/g dw) and Predicted responses (mg/g dw): C_a_; Chlorophylle a. C_b_; Chlorophylle a. C_c1+c2_; Chlorophylle c.

**Table 2:** Central Composite Design for the Optimization of Total Chlorophyll, Carotenoids, and Biomass Yield in *Dunaliella tertiolecta*.

	Experimental Variables				Responses (mg/g dw)			Predicted Responses (mg/g dw)		
Run	Pattern	*X*_1_	*X*_2_	*X*_3_	T-Chl	T-Car	Yield	T-Chl	T-Car	Yield
1	0A0	0.55	45	10	3.07	0.99	0.133	2.10	0	0.13
2	00a	0.55	35	5	1.89	0.11	0.1	2.91	0	0.10
3	a00	0.22	35	10	3.83	1.19	0.065	6.72	2.43	0.05
4	−++	0.22	45	15	1.26	0.72	0.108	3.6	1.73	0.12
5	0a0	0.55	25	10	3.74	0.86	0.1	7.66	2.68	0.10
6	00A	0.55	35	15	1.48	0.53	0.166	3.42	1.38	0.16
7	000	0.55	35	10	2.34	0.76	0.101	2.43	0.07	0.11
8	++−	0.88	45	5	13.35	2.78	0.023	15.35	4.59	0.02
9	+−+	0.88	25	15	4.96	0.72	0.127	6.78	1.45	0.13
10	−−+	0.22	25	15	23	13.26	0.06	22.8	11.37	0.06
11	+−−	0.88	25	5	7.8	1.77	0.038	7.26	0.58	0.03
12	+++	0.88	45	15	2.96	0.89	0.101	4.69	0.18	0.1
13	−−−	0.22	25	5	11.04	3.55	0.018	11.11	4.08	0.02
14	A00	0.88	35	10	5.28	0.46	0.028	5.34	0	0.04
15	−+−	0.22	45	5	2.12	0.62	0.1	2.1	0	0.1

Experimental variables as the independent variables: X_1_, nitrogen concentration (mol·L^−1^ NaNO_3_). X_2_, Salinity (PSU). X_3_, Culture age (days). Responses (mg/g dw) and Predicted responses (mg/g dw): T-Chl, Total Chlorophylle. T-Car, Total carotenoids, Yield.

**Table 3:** Pigment profile of *Dunaliella tertiolecta* extract by HPLC at 457 nm.

Peak	Name	Retention Time (min)	Relative Abundance (%)
1	all-trans violaxanthin	1.65	45.16
2	13-cis lutein	2.12	0.5
3	all-trans luteoxanthin	2.98	2.7
4	all-trans zeaxanthin	4.01	2.43
5	all-trans α-carotene	5.52	3.6
6	all-trans β-carotene	6.25	4.5
7	9-cis-β-carotene	9.08	34.32

**Table 4:** Characterization of Dt-LN and β-CAR-LN: encapsulation efficiency (EE, %), z-average diameter (nm), PDI, and zeta potential (mV). Data represent the mean value ± standard deviation (*n* = 3).

Formulation	EE (%)	Particle Size (nm)	Polydispersity Index (PDI)	Zeta Potential (mV)
Dt-LN	63.78 ± 2.24	387 ± 21.32	0.26 ± 0.057	−32 ± 0.97
β-CAR-LN	94.67 ± 3.56	320 ± 12.92	0.33 ± 0.037	−24.23 ± 1.23

**Table 5:** Antioxidant activity assessed by ABTS^•+^ and FRAP assays for nanoliposomes loaded with *Dunaliella tertiolecta* extract, β-carotene, unloaded nanoliposomes, microalgal extract, and commercial β-carotene.

Sample	ABTS^•+^ (µmol TE/g)	FRAP (µmol TE/g)
*Dt*-NL	12.44 ± 0.12 ^c^	46.11 ± 0.83 ^c^
β-CAR-NL	10.8 ± 0.17 ^b^	41.52 ± 1.14 ^b^
NC-NL	0 ^a^	0 ^a^
Microalgal extract	12.92 ± 0.11 ^d^	47.62 ± 0.88 ^cd^
β-Carotene	12.41 ± 0.16 ^c^	49.45 ± 0.6 ^d^

Different letters within the same column indicate significant differences between treatments at *p* < 0.05.

**Table 6:** Erythroprotective potential of nanoliposomes loaded with *Dunaliella tertiolecta* extract and β-carotene against oxidative damage on human erythrocytes.

	Hemolysis Inhibition (%)			
Sample	A^+^	B^−^	O^+^	AB^+^
***Dt*-LN**	53.17 ± 4.66 ^b^	49.67 ± 3.79 ^b^	54.84 ± 8.82 ^b^	45.53 ± 7.6 ^c^
**β-CAR-LN**	50.04 ± 10.42 ^b^	47.11 ± 6.17 ^b^	49.63 ± 10.5 ^b^	39.03 ± 6.5 ^bc^
**UL-N**	8.72 ± 0.42 ^a^	8.54 ± 0.34 ^a^	8.4 ± 0.4 ^a^	8.56 ± 0.33 ^a^
**Microalgal extract**	46.62 ± 4.66 ^b^	51.22 ± 2.06 ^b^	46.45 ± 4.6 ^b^	28.12 ± 4.62 ^b^
**β-carotene**	94.70 ± 3.83 ^c^	75.55 ± 10.89 ^c^	94.66 ± 3.86 ^c^	47.69 ± 1.67 ^c^

Different letters within the same column indicate significant differences between treatments at *p* < 0.05. Dt-LN = *Dunaliella tertiolecta*-Loaded Nanoliposome; β-Car-LN = β-Carotene-Loaded Nanoliposome; UL-N = unloaded nanoliposomes.

**Table 7:** Cytotoxicity (%) for nanoliposomes loaded with *Dunaliella tertiolecta* extract and β-carotene against oxidative damage on human erythrocytes.

	Cytotoxicity (%)			
Sample	A^+^	B^−^	O^+^	AB^+^
***Dt*** **-LN**	17.22 ± 3.31 ^c^	20.46 ± 4.39 ^b^	21.62 ± 4.52 ^b^	6.59 ± 1.01 ^a^
**β-CAR-LN**	17.48 ± 3.07 ^c^	14.94 ± 1.56 ^a^	15.92 ± 1.61 ^a^	22.25 ± 3.76 ^d^
**UL-N**	11.22 ± 2.21 ^b^	12.34 ± 2.25 ^a^	13.24 ± 2.04 ^a^	10.57 ± 1.47 ^b^
**Microalgal extract**	20.47 ± 1.79 ^c^	22.47 ± 1.98 ^b^	23.68 ± 2.04 ^b^	15.33 ± 1.87 ^c^
**β-carotene**	2.36 ± 0.85 ^a^	13.88 ± 1.91 ^a^	14.83 ± 1.95 ^a^	5.7 ± 0.69 ^a^

Different letters within the same column indicate significant differences between treatments at *p* < 0.05. Dt-LN = *Dunaliella tertiolecta*-Loaded Nanoliposome; β-Car-LN = β-Carotene-Loaded Nanoliposome; UL-N = unloaded nanoliposomes.

**Table 8:** Functional groups identified in the loaded nanoliposomes, microalgal extract, and β-carotene using Fourier-transform infrared spectroscopy (FT-IR).

Functional Group	Band	Wavenumber (cm^−1^)	Present in
**Alkynes**	C-H (acetylenic)	~3300	Microalgal extract
**Aromatic**	C-H	~3020–3000	All
	C=C	~1600	*Dt*-NL and microalgal extract
**Alcohols**	O-H	~3650–3300	All
**Carboxylic acids**	O-H	3400–2400	*Dt*-NL and microalgal extract
	C=O	1730–1700	NC-NL and *Dt*-NL
**Amines**	N-H	3500–3180	*Dt*-NL and microalgal extract
	C-N	~1200	*Dt*-NL and microalgal extract
**Amides**	N-H	~1640	*Dt*-NL and microalgal extract
**Alkanes**	C-H	2950–2800	All
	C-H_2_	~1450	All
**Alkenes**	C=C (Isolated)	~1690	All
	C=C (Conjugated)	~1640	*Dt*-NL and microalgal extract
	C-H	~1430	All
**Esters**	C=O	1750–1735	NC-NL, β-CAR-NL and β-carotene
**Nitro groups**	-NO_2_ (aliphatic)	~1390	*Dt*-NL and microalgal extract

**Table 9:** A completely random factorial design of the treatments (nitrogen concentration and different salinities) was applied in the microalgae culture.

Nitrogen Concentration	Salinity (PSU)
0.88 mol·L^−1^ (1) *	25
	35
	45
0.44 mol·L^−1^ (0.5) *	25
	35
	45
0.22 mol·L^−1^ (0.25) *	25
	35
	45

* mL de NaNO_3_ for each litter of seawater for culture.

## Data Availability

The data supporting the findings of this research are provided within this article. Additional details can be obtained from the corresponding authors upon request.

## Supplementary Materials

The following supporting information can be downloaded at: https://www.mdpi.com/article/10.3390/molecules30193924/s1, Figure S1: HPLC chromatograms of the extract of *Dunaliella tertiolecta*.
