# Supplementary material for: Nanoencapsulated Dunaliella tertiolecta Extract and β-Carotene in Liposomal Carriers: Antioxidant and Erythroprotective Potential Through Sustained-Release Systems
Source: Molecules. 2025 Sep 29;30(19):3924. doi: 10.3390/molecules30193924 (PMC12525773; doi:10.3390/molecules30193924)
Supplement: Supplementary file 1 [file molecules-30-03924-s001.zip › molecules-3831740-supplementary.pdf]

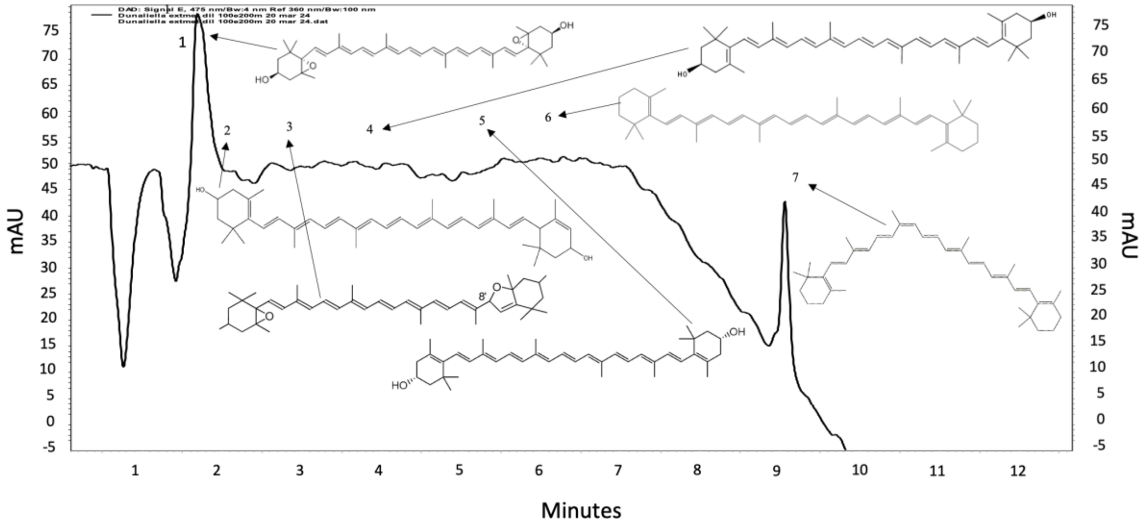

Supplementary material Figure S1. HPLC chromatograms of the extract of *Dunaliella tertiolecta*. X axes represents retention time in minutes, Y axes represents detector response at 457 nm (mAU). Peak 1 is all-trans violaxanthin, peak 2 is 13-cis lutein, peak 3 is all-trans luteoxanthin, peak 4 is all-trans zeaxanthin, peak 5 is all-trans α-carotene, peak 6 is all-trans β-carotene and peak 7 is 9-cis-β-carotene.
